# Public Health Interventions Delivered by Pharmacy Professionals in Low- and Middle-Income Countries in Africa: A Systematic Scoping Review

**DOI:** 10.3390/pharmacy11010024

**Published:** 2023-01-30

**Authors:** Begashaw Melaku Gebresillassie, Kelly Howells, Diane Ashiru-Oredope

**Affiliations:** 1 School of Pharmacy, University of Gondar, Gondar P.O. Box 196, Ethiopia; 2 School of Health Sciences, The University of Manchester, Manchester M13 9PL, UK; 3 Centre for Women’s Health Research, School of Medicine and Public Health, The University of Newcastle, Newcastle, NSW 2300, Australia; 4 School of Pharmacy, University of Nottingham, Nottingham NG7 2RD, UK

**Keywords:** pharmacist, pharmacy technician, health promotion, health protection, health improvement, interventions

## Abstract

Pharmacists and their teams play an important role in providing public health services, however little is known about their level of contribution and the strength of evidence in Africa’s Low- and Middle-Income Countries (LMICs). The purpose of this scoping review was to explore and map the available evidence on pharmacy professional-delivered public health interventions in Africa’s LMICs. Six electronic databases (Medline, Embase, International Pharmaceutical Abstract, PsycInfo, Maternity and Infant Care, and Cochrane database), relevant grey literature sources, key journals focused on African health issues, and libraries of relevant organizations were searched between January 2010 and December 2020. Studies were included if they reported public health interventions delivered by pharmacy professionals (pharmacists or pharmacy technicians) or their teams. The quality of the individual studies was assessed using an adapted grading system. Thirty-nine studies were included in this review. Pharmacy professionals delivered a wide range of public health interventions, with the most common themes being noncommunicable diseases, infectious diseases, sexual and reproductive health, antimicrobial resistance, and other health conditions, e.g., dental health, unused drugs or waste, minor ailments. The majority of the studies were classified as low-quality evidence. They were predominantly feasibility and acceptability studies conducted in a narrow study area, in a small number of LMICs in Africa, resulting in little evidence of service effectiveness, issues of broad generalizability of the findings, and sustainability. The major constraints to service provision were identified as a lack of training, public recognition, and supporting policies. Pharmacy professionals and their teams across LMICs in Africa have attempted to expand their practice in public health. However, the pace of the expansion has been slow and lacks strong evidence for its generalizability and sustainability. Future research is needed to improve the quality of evidence, which will subsequently serve as a foundation for policy reform, allowing pharmacy professionals to make significant contributions to the public health initiatives in the region.

## 1. Introduction

Africa has the largest disease burden (per population) of any continent, as well as the smallest quantity and ratio of health workforce [1,2]. The continent, in particular, is suffering from a double burden of diseases: in addition to continuing high rates of infectious diseases such as malaria, human immunodeficiency virus (HIV)/acquired immunodeficiency syndrome (AIDS), tuberculosis (TB), and other infections, cardiovascular diseases (CVDs) and diabetes are among the chronic noncommunicable diseases (NCDs) rising rapidly in the region. As a result, the major causes of mortality have shifted from infectious diseases to a combination of infectious and NCDs [3,4,5,6]. As core members of one of most accessible health care providers/premises, pharmacists and pharmacy technicians, hereafter known as pharmacy professionals, have an important role in the areas of public health practice [7]. The majority of the health workforce delivering public health interventions in Africa are physicians, nurses, and community health workers, targeting at a variety of populations, including maternal and child health, infectious disease control, and non-communicable diseases in Africa [8]. For example, physicians and nurses are typically responsible for providing clinical care, such as diagnosing and treating illnesses. Community health workers, on the other hand, are responsible for outreach and education, as well as for connecting individuals to healthcare services [8]. In the health improvement area of practice, pharmacy professionals and their teams can provide support in improving the health and well-being of the population, can reduce health inequalities by proactively promoting health and well-being messages, and can support and enable people to adopt healthier lifestyles, take responsibility for their own and their families’ health, and support self-care [7].

The health protection domain is concerned with protecting the population’s health and well-being by helping in the prevention and transmission of communicable and other infectious diseases, screening for risk factors and disease, ensuring prudent antibiotic use, supporting in the mitigation of antimicrobial resistance risks, protecting against pharmaceutical hazards, and supporting the pharmacy response to an emergency [7]. This is a diverse role, and pharmacy professionals and their teams can contribute to and promote antimicrobial stewardship programs; participate in early screening, treatment, and referral programs; collaborate with other healthcare professionals to improve immunization uptake for at-risk patients; ensure the safe storage, administration and disposal of all medicinal products; and effectively communicating information on threats to health to patients and the public [7]. The area of health service delivery and quality is concerned with efforts aimed at improving the service quality [7,9]. Innovative, high-quality pharmacy public health services improve health outcomes, while also ensuring equitable and effective resource allocation. Pharmacy professionals and their teams can plan services, undertake audits, and evaluate services to improve the service quality [7,9]. They can, for example, conduct audits to improve the prescription quality and safety or monitor treatment guideline adherence to improve patient safety by ensuring the safe use of high-risk medications, such as warfarin [7]. They can also inform the general public on the range of public health services available from pharmacies, as well as any restrictions on service availability, in formats that are accessible to a wide range of people [7].

In 2006, the International Pharmaceutical Federation (FIP) issued a policy statement on the role of pharmacists in the prevention and treatment of chronic diseases [10]. More recently, in 2019, the FIP approved an expanded role of pharmacists, including prevention and screening activities, patient referral when appropriate, and pharmacy professional-led, patient-centered noncommunicable diseases (NCDs) management to improve the outcomes and quality of life [11]. This working document described pharmacists’ involvement and intervention in different areas of NCDs. There have been several cases in which pharmacy professionals’ involvement in NCD management has resulted in improved treatment outcomes, better adherence to treatment and a healthy lifestyle, and fewer medication-related issues [12,13].

As the global agenda also shifts towards addressing the Sustainable Development Goals (SDGs), pharmacy professionals are primed to collaborate with others to help strengthen pharmaceutical systems in order to achieve the third SDG of good health and well-being for all. In this regard, organizations such as the World Health Organisation (WHO), FIP and Commonwealth pharmacists’ association recognized the need and potentially significant contribution of pharmacy professionals to improve the public’s health [10,11,14]. The recent Coronavirus Disease 2019 pandemic has also revealed that Africa, in particular, needs a new public health approach to be resilient and to adapt and cope with 21st century disease threats. The suggested new approach emphasizes adequate investment to develop and maintain a prepared cadre who will be fundamental for the continent to cope with its disease threats [2]. As learned from the Ebola crisis, resilient health systems function and respond best when there is clarity regarding the roles of the different actors within the system, including that of pharmacy professionals [15].

There is a growing recognition that pharmacy professionals are utilizing their medication expertise by moving away from the dispensing window and demonstrate the value of the years invested in pharmacy schools to improve the health and well-being of communities [16,17,18]. For example, in Nigeria and Ghana, they are screening patients for NCDs such as hypertension and diabetes and advising them on lifestyle management [19,20]. In Sudan, pharmacy professionals are providing tobacco cessation, diet, contraception, lifestyle counseling, and weight reduction interventions [21], and in Kenya, Sudan and Uganda, they have provided sexual and reproductive health services including distribution and advice around different contraception methods for several years [21,22,23,24]. Furthermore, pharmacy professionals are becoming involved in maternal and child health services in Ethiopia and Uganda [18,25,26]. Enabling pharmacy professionals to deliver these health services at scale would have a significant advantage from different perspectives for LMICs in Africa, such as improving patients’ access to health care and alleviating the burden on other health providers, including physicians and nurses, allowing them to focus on patients with acute care needs.

Findings have indicated that there is a variation in pharmacy professionals’ responses for managing infectious and non-infectious conditions, as well as sexual and reproductive health services, which is impacted by different levels of pharmacy professionals’ knowledge, views, and opinions in various public health services [19,21,23,26,27]. Moreover, despite these studies being conducted across several countries, including Ghana [20], South Africa [28], Sudan [21], Nigeria [19], Kenya [23], Ethiopia [26], and Tanzania [27], there is paucity in the literature focused on the synthesis of these findings. In order to continue to improve the contribution of pharmacy professionals across the public health areas, summarized information is needed regarding the involvements, type, and extent of public health interventions provided by pharmacy professionals. Therefore, the main goal of this review was to explore and map the available evidence from the perspective of pharmacy professionals, public health interventions, and LMICs in Africa.

One of the main goals of the agenda set by the WHO for the future of public health is to form accessible, multidisciplinary networks of public health professionals who actively engage within communities and provide critical public health services [29,30]. Pharmacy professionals have the knowledge, skills, and vast opportunities around the continent to play a role in facilitating and providing quality public health services, as evidenced in the reports by the FIP [10,11,31]. There is a scarcity of evidence on the extent to which pharmacy services are used in everyday practice and their influence on public health. In this regard, what is known about the public health role of pharmacy professionals is from studies conducted in developed countries [17,32,33,34], with a clear evidence gap focused on the synthesis of these findings in LMICs in Africa.

The principal aim of this scoping review was to explore and map the available evidence on pharmacy professional-delivered public health interventions in Africa’s LMICs (Supplementary File S1), and to provide the key stakeholders, such as researchers, practitioners, policymakers, and the general public, with information on the range of services investigated and the areas in which additional research is needed, as well as the characteristics of the evidence and potential barriers to service provision. The research question that guided this review was: What is known about the contribution of pharmacy professionals in Africa’s LMICs to public health and the nature of evidence?

## 2. Methods

This scoping review project was conducted between February 2021 to July 2021 and followed the guideline provided by the Preferred Reporting Items for Systematic Review and Meta-Analysis Extension for Scoping Reviews (PRISMA-ScR) [35]. The completed reporting checklist is available in Supplementary File S2.

### 2.1. Search Strategy and Information Sources

A systematic search of the evidence that involved searching for existing literature via different sources was adopted. The complete search strategy is presented in Supplementary File S3.

### 2.2. Electronic Search

The search strategy for the electronic databases was developed by breaking down the research question using the PIPOH (Population, Interventions, Professionals, Outcomes, Health care setting) framework, as recommended by the Joanna Briggs Institute (JBI), and defining the key concepts [36]. The search terms or keywords, including the Medical Subject Heading (MeSH), for the public health interventions were defined and adapted from the Royal Pharmaceutical Society’s Professional Standards for Public Health Practice for Pharmacy guideline [7]. Terms for pharmacy professionals and medication retail outlets were developed by literature search [37,38]. The initial or piloting search was conducted to allow for refinement based on the early results. The final search strategy was first used on the Medline database and then converted for each successive database (Embase, International Pharmaceutical Abstract, PsycInfo, Maternity and Infant Care Database, and Cochrane database of systematic review). These six databases were selected to ensure all of the relevant literature were identified systematically and also to prevent selection and retrieval bias.

### 2.3. Hand-Searching of Key Journals, Theses and Dissertations, and Relevant Organizations

Ten common journals (Supplementary File S3) that focused on health issues of the African continent were hand-searched. The hand searching involved a manual page-by-page investigation of the entire contents of the journals’ issues or conference proceedings to identify all of the eligible reports. Similarly, hand searching was also conducted on EBSCO Open dissertations, Networked Digital Library of Theses and Dissertations (NDLTD) global Electronic Theses and Dissertation (ETD) search, and BIELEFELD Academic Search Engine (BASE) to identify theses and dissertations related to the gray literature. Four references were located by this searching technique and were included for screening. The searching was also conducted by identifying the relevant local or international organizations working in the field, with a view to hand-search their libraries to identify either published and/or unpublished work.

Studies were eligible for inclusion if they were: (i) undertaken following either interventional (e.g., randomized and non-randomized controlled trials, before and after intervention studies) or observational designs (e.g., cross-sectional, cohort, or case-control studies); (ii) conducted in Africa’s low- and middle-income countries; (iii) conducted between 2010 and 2020, as the literature has grown in recent years, and the FIP has expanded the scope of pharmacy practice to incorporate public health since 2006, in collaboration with the WHO [10]; (iv) focused on health improvement, health protection, and healthcare public health; (v) services provided by licensed pharmacy professionals or medicine retail outlets, such as pharmacies, drug stores, drug vendors or shops, accredited drug dispensing outlets, and licensed chemical seller shops. In addition, any existing literature on pharmacy public intervention were included, including reviews, organizational reports, theses, dissertations, conference papers, commentaries, and case studies. The detailed inclusion and exclusion criteria were presented in the Supplementary File S1.

### 2.4. Data Extraction

The screening and eligibility assessment of the articles from the literature search was conducted in a standardized manner by BMG and DAO; the literature search, screening and eligibility assessment were conducted by BMG and the random literature search on the works initially classified as uncertain was conducted by DAO, and the conflict was resolved through discussion. The search results from each database were imported to the Endnote version 20 reference manager software, and the screening was conducted in two steps.

Step 1: Following the removal of duplicates, the studies were screened based on their titles and abstracts, using the inclusion and exclusion criteria 1–9. Studies that fulfilled the predetermined criteria were excluded and recorded as irrelevant. If the abstracts were unavailable and the study was potentially relevant, the full text was sought.

**Figure 1:**
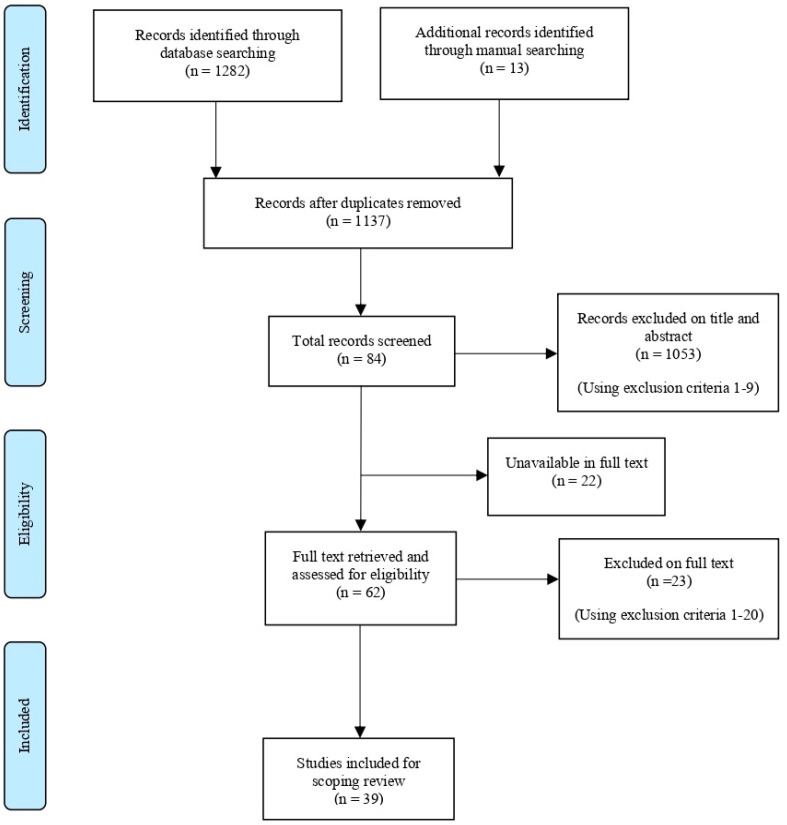
PRISMA-ScR flow chart of the study selection process.

Step 2: Full reports of the potentially relevant studies, according to the inclusion criteria, were retrieved and a detailed screening using the exclusion criteria 1–21 was undertaken. A flow chart (Figure 1) was used to document the decisions made at each stage of the screening process.

### 2.5. Data Charting

Charting or sorting the data according to key themes is a technique for the synthesis and interpretation of qualitative data [35]. A data-charting form was jointly developed by three of the authors to determine which variables to extract. BMG and DAO charted the data during live online discussions, discussed the results, and continuously updated the data charting form in an iterative process. The following data were gathered for the charting exercise and served as the foundation for the analysis:

Items used to describe the study context:

- the country setting (e.g., LMICs in Africa),
- the setting type (e.g., community pharmacy, hospital pharmacy, health center, hospital),

Items used to describe the intervention of interest:

- the intervention type (e.g., advice, testing, monitoring),
- the health condition (e.g., diabetes, hypertension, Asthma, malaria),

Items used to describe the study design:

- The aims of the study and the aims of the intervention,
- The type of study design (e.g., randomized controlled trial (RCT), quasi-experimental, single-arm before and after intervention, quantitative cross-sectional survey, qualitative study, mixed-method design,
- Outcomes and processes assessed,
- The sample size,
- Characteristics of the sample (e.g., gender, age, health status),
- Resource investment (e.g., training),
- Mode of delivery,
- The number of required interactions to deliver the intervention,

### 2.6. Collating, Summarizing, and Reporting the Findings

Following the charting of the information from the included studies, the narrative account of the findings was presented in two ways. The first section focused on the numerical analysis of the publication rates, study distribution, and health conditions covered. As a result, the chart mapping was developed to describe the rate of publication, the geographic distribution of the studies, and the range of health conditions addressed. This has assisted in quickly grasping the main areas of service provision. The second section focused on thematically organizing the literature [35] in accordance with the five key public health intervention themes identified in the scoping review. This made the public health intervention themes the primary unit of analysis for organizing the final result. In addition, the quality of the evidence was assessed by assigning an evidence grade to each identified study using a grading system adapted from the public health literature [39,40,41]:

Level A: Evidence from meta-analysis or systematic reviews of randomized controlled trials.
Level B: Evidence from randomized controlled trials.
Level C: Evidence from quasi-experimental or intervention studies
Level D: Evidence from observational studies or quantitative surveys or well-designed qualitative studies
Level E: Expert opinion, case reports.

## 3. Results

### 3.1. Study Selection

A total of 1282 records were identified through electronic database searching, and 13 additional records were identified through a manual hand search. The records were retrieved from six electronic databases. After removing 158 duplicates, 1137 studies were screened for their title and abstract. In total, 1053 records were excluded through the assessment of the titles and abstracts for relevance, and 84 full-text studies were further assessed against the inclusion and exclusion criteria (Supplementary File S1). Following a detailed evaluation of these studies, 39 full-text studies were included for the final analysis. Figure 1 shows the flow of studies identified by the searches.

### 3.2. Study Characteristics

**Figure 2:**
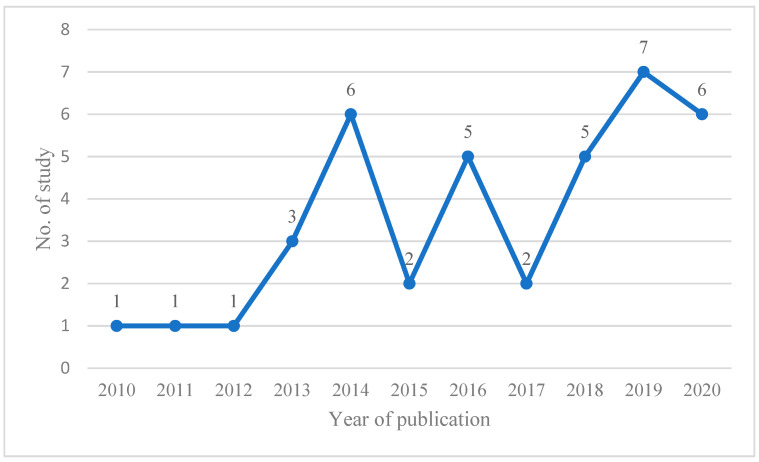
The rate of publication of papers describing pharmacy professionals’ public health interventions in Africa.

To highlight how the public health involvement of pharmacy professionals has progressed in the last decade, following the release of the pharmacy-centered policy recommendations from the WHO and FIP, the rate of publication of research papers in the area is provided in Figure 2. The lowest number of studies examining pharmacy professionals’ contributions in the selected areas of public health was found between the years 2010 and 2013. The rate of publication first peaked in 2014 (*n* = 6). After 2018, the number of published studies was high, and reached the second peak in 2019 (*n* = 7) (Figure 2).

**Figure 3:**
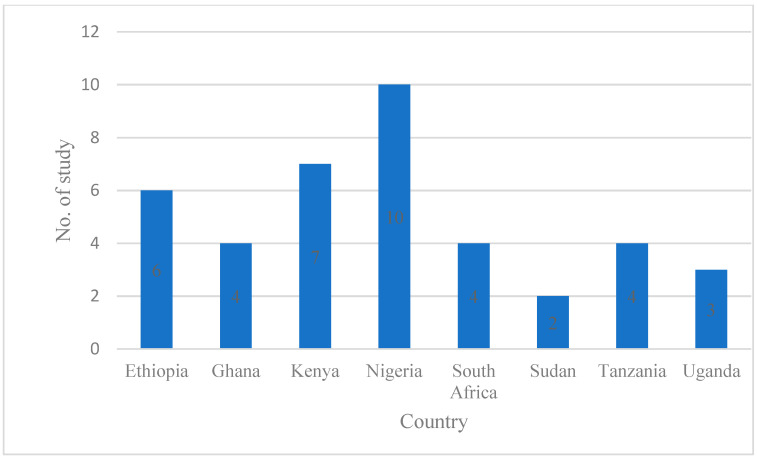
The geographical distribution of included studies, with one study conducted in both Kenya and Nigeria (*n* = 40).

For this scoping review, studies were included only if they were reporting pharmacy professionals’ interventions within the low- and middle-income countries of Africa [42]. Of the included 39 studies, the majority (10; 25%) were conducted in Nigeria, followed by Kenya (7; 17.5%), and Ethiopia (6; 15%). The remaining studies were conducted in Ghana, Tanzania, South Africa, Uganda, and Sudan, each with less than five studies (Figure 3).

### 3.3. Scope of Public Health Interventions and Quality of Evidence

The scoping review found that pharmacy professionals were providing a wide range of public health services, with the dominant themes being:

- Non-communicable diseases (NCDs) (*n* = 15),
- Infectious diseases (*n* = 12),
- Antimicrobial resistance (*n* = 5),
- Sexual and reproductive health (*n* = 11),
- Other health conditions (*n* = 5)

**Figure 4:**
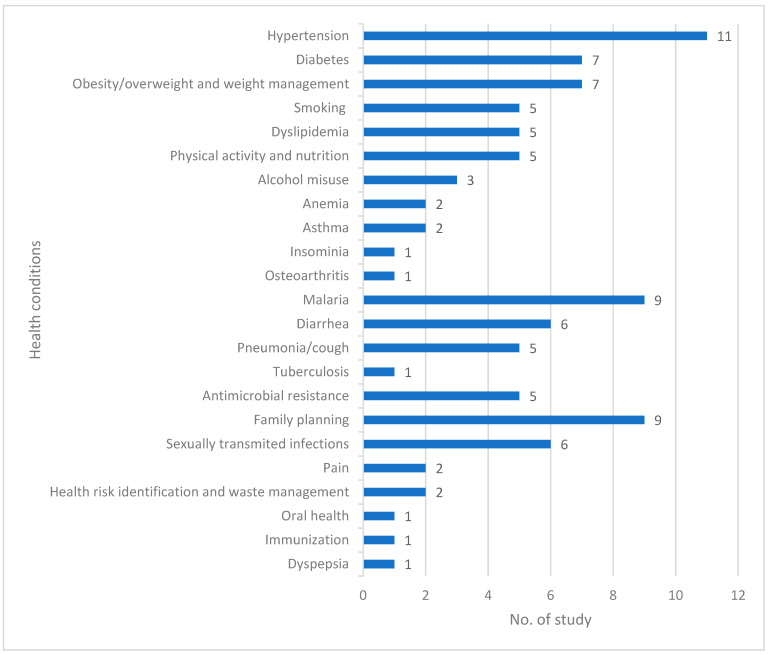
Health conditions covered by the included studies, with 14 studies addressing multiple conditions.

The majority of the included studies assessed the service provision of pharmacy professionals for hypertension (*n* = 11), malaria (*n* = 9), and family planning (*n* = 9) health conditions. The second-largest group of studies investigated the contribution of pharmacy professionals in the areas of diabetes (*n* = 7), obesity and weight management (*n* = 7), diarrhea (*n* = 6), and sexually transmitted infections (*n* = 6). Other key health conditions investigated include pharmacy professionals’ service provision to address smoking (*n* = 5), dyslipidemia (*n* = 5), physical activity and nutrition (*n* = 5), pneumonia (*n* = 5), antimicrobial resistance (*n* = 5), and three studies addressed alcohol abuse or misuse (Figure 4). In fourteen of the studies, more than one health issue was addressed. Full details on the types of studies included, the aims of the interventions, the study settings and design, the intervention component, the mode of delivery and the training are presented in Supplementary File S4.

#### 3.3.1. Noncommunicable Diseases (NCDs)

There were fifteen studies identified across six countries that explored the service provision of pharmacy professionals in the prevention and control of NCDs [18,19,20,21,28,43,44,45,46,47,48,49,50,51,52]. Diabetes, hypertension, obesity, weight management, dyslipidemia, physical inactivity, an unhealthy diet, smoking, and alcohol misuse were among the health conditions primarily targeted by the interventions. The service delivery primarily involved detecting and lowering cardiovascular risk factors, as well as disease state management. The studies used a range of study designs, such as cross-sectional, single-blind randomized controlled trials, quasi-experimental designs, and single group before and after intervention studies (Table 1 and Supplementary File S4)

#### 3.3.2. Infectious Disease

A total of twelve studies, from seven countries, that examined the service provision of pharmacy professionals in the infectious disease theme were identified [25,26,27,53,54,55,56,57,58,59,60,61]. Malaria, diarrheal disease, and tuberculosis were the primary targets of the interventions. The primary focus of the service delivery was on testing, treating, and referring conditions. Among the study designs used were a cross-sectional study with a mixed-method approach combining survey and qualitative interview, a quasi-experimental, a cluster-randomized trial, and a randomized interventional design (Table 1 and Supplementary File S4).

#### 3.3.3. Antimicrobial Resistance

A total of five studies from three countries were identified that explored the service provision of pharmacy professionals in the antimicrobial resistance theme [18,62,63,64,65]. The interventions were largely designed to evaluate antibiotic stewardship interventions. Antimicrobial therapy review and auditing, protocol development, feedback, and in-service education for antimicrobial use were all provided. The studies were conducted utilizing a cross-sectional and single group before and after intervention design (Table 1 and Supplementary File S4).

#### 3.3.4. Sexual and Reproductive Health

Eleven studies were found from six countries that explored the service provision of pharmacy professionals in the sexual and reproductive health theme [18,21,22,23,24,48,66,67,68,69,70]. Sexually transmitted infections (STIs), including HIV/AIDS, and family planning were the primary targets of the interventions. The screening and treatment of STIs, as well as family planning and referral services, counselling, and education, were provided. The studies were carried out using a variety of study designs, including cross-sectional, qualitative, and interventional designs (Table 1 and Supplementary File S4).

#### 3.3.5. Other Health Conditions

Five studies, from three countries, were found that examined pharmacy professionals’ service provision for a variety of health conditions not covered by the preceding four themes [18,21,43,48,61]. The interventions focused on back pain, dyspepsia, immunization, dental health, unused drugs or waste, minor ailments, and identifying community health risks. Vaccine distribution, need assessments, counseling, diagnosis and treatment of conditions, and physician referrals were all provided. The studies were conducted using a cross-sectional design (Table 1 and Supplementary File S4).

### 3.4. The Strength and Level of Evidence

The included 39 studies in this scoping review had various levels of evidence, ranging between B and D. (Supplementary File S4). The majority of the studies (*n* = 25) generated level D evidence (observational studies, quantitative surveys or well-designed qualitative studies); twelve had level C evidence (quasi-experimental or intervention studies); and only two generated level B evidence (randomized controlled trials). The level B studies involved randomized controlled trials examining the effectiveness of rapid diagnostic tests for malaria detection in pharmacies [58] and pharmacy professional-led interventions on an asthma management and adherence program [52]. The level C evidence centered on the pharmacy professional-led screening and management of infections and NCDs [20,25,28,45,51,54,55], antimicrobial stewardship programs [62,63,64,65], and emergency contraception supply [23]. The majority of the studies generating level D evidence utilized surveys, with or without mystery patient methodology, to examine the actual practice of pharmacy service provision, while a few used qualitative interviews or focus group discussions with mystery patient methodology [18,19,21,22,24,26,27,43,44,46,47,48,49,50,53,56,57,59,60,61,66,67,68,69,70]. One-quarter of the included studies (*n* = 9) examined processes such as acceptability and feasibility of specific services [20,25,44,45,47,54,59,68,70]. Almost half of the studies (*n* = 17) reported that public health services offered by pharmacy professionals were of poor quality and had low success rates, indicating structural and institutional barriers to their implementation [18,19,21,23,24,26,47,48,49,50,53,60,61,65,66,67,69]. In three studies focusing on infectious diseases, there was little involvement of pharmacy professionals in malaria prevention programs and appropriate diarrhea management [26,53,61]. In four studies on the sexual and reproductive health theme, low service quality was reported, including inconsistent counseling and incorrect information regarding family planning methods, as well as a failure to comply with national guidelines during STI management [23,66,67,69]. Similarly, lower levels of participation in NCDs screening, treatment, and referral, vaccination supply, and conducting needs assessments to identify community health risks were reported in the NCDs, infection and antimicrobial resistance themes [18,19,21,47,48,49,50,60,65]. Lack of health promotion activities in pharmacies, lack of staff or resources, lack of knowledge, lack of training, lack of confidence, lack of public recognition, lack of collaboration with other health care professionals, and lack of supportive policies were the reported barriers to provide appropriate services [18,19,21,23,24,47,48,49,50,53,60,61,65,66,67,69].

## 4. Discussion

Whilst there have been reviews focused on high income countries [38,71], to the authors’ knowledge, this is the first scoping review to investigate pharmacy professionals’ contributions to public health across multiple countries in Africa. This review highlighted pharmacy professionals’ involvement in public health services, indicating that their roles are expanding beyond dispensing. To increase the breadth of service provision, interventions delivered by all types of licensed medicine retail outlets and pharmacy professionals were included. The licensed medicine retail outlets consisted of pharmacies, drug stores, drug vendors or shops, accredited drug dispensing outlets, and licensed chemical seller shops. They were run by licensed pharmacy professionals with different qualifications. The identification and evaluation of the present public health pharmacy services uncover the current opportunities and possible future development areas. The findings also revealed several implementation challenges, as well as the main reasons why they were not implemented appropriately, adequately, or consistently in the community.

The main finding of this review was that pharmacy professionals provided a variety of public health services across LMICs in Africa, and these services were grouped into five different categories (noncommunicable diseases, infectious diseases, sexual and reproductive health, antimicrobial resistance, and other health conditions). The majority of the included studies investigated pharmacy professionals’ service delivery in the areas of NCDs, infectious diseases, antimicrobial resistance and sexual and reproductive health. The studies investigated interventions for identifying individuals who are at risk of developing NCDs, treating people who have NCDs [18,19,20,21,28,43,44,45,46,47,48,49,50,51,52], testing, treating, and referring people with common infectious conditions, antibiotic stewardship, and family planning services. Furthermore, the intervention addressed six additional health conditions not covered by the preceding themes, including dyspepsia, oral health, pain, immunization, unused medicine or waste management, and community health risk identification. This indicated that pharmacy services in Africa’s LMICs are moving in the same direction as the global context and following the trends of developed countries, with service expansion from conventional dispensing activities to increasing engagement in health improvement, health protection, and service improvement areas [10,11,72].

In the majority of the included studies, identifying the target population was often difficult as the nature of medicine retail outlets is to serve a local or general population in their catchment area, and encounters were often opportunistic. However, in several NCDs studies that focused on identifying and reducing the risk factors for cardiovascular disease, the intervention was directed towards adults and older people [20,28,43,44,45,46,47]. In malaria, pneumonia, and diarrhea studies, the service provision was primarily targeted at children under the age of five or people living in hard-to-reach areas [25,26,55,56]. Similarly, in certain sexual and reproductive health studies, services were provided to young people who were sexually active and planned to use various family planning methods [66,69].

Although the different types of staff training undertaken to provide the services were not reported in all of the included studies, the pharmacy professionals were offered face-to-face training on how to measure blood pressure, blood glucose, body mass index (BMI), waist circumference, mobile health app use, and patient referral procedures in the NCDs studies [20,28,44,45,47]. They were also trained in the appropriate testing and treatment of malaria, pneumonia, and diarrhea in the infectious disease studies. The training covered disease signs and symptoms [54,55,58,59], how to perform Rapid Diagnostic Testing [25,54,57,59], how to take a blood sample and make a blood slide [58], blood safety, and sharp objects handling [58], infection prevention procedures [58], referral of patients [54,58,59], treatment with combination drugs [25,54,55,57,58,59], and malaria drug policy [58]. In the sexual and reproductive health studies, they were trained in client engagement [70], provision of family planning methods [22,23,68], referring clients for injection [68,70] or other methods of contraception or complications [22,23], recording of service utilization [68], and collecting client information [68], using a variety of learning methodologies, including demonstration, group discussion, and role-play simulations. The training sessions were held both on-site at the pharmacy setting and, on occasion, off-site. Similarly, they were also given in-person instruction on how to perform antibiotic usage audits and provide input on antimicrobial resistance studies [62,64]. This emphasizes the value of capacity-building training in improving professionals’ knowledge, skills, self-efficacy, and practice or policy changes.

The majority of the included studies were not designed to provide high-quality evidence, implying that much of the evidence is still proof-of-concept rather than knowledge translation. In this aspect, RCT-tested interventions for improving practice had a high success rate [41]. This may be due to the nature in which medicine retail outlets operate, which makes conducting randomized controlled trials difficult. In the studies that focused on NCDs, only three of the thirteen studies were interventional studies; one adopted a quasi-experimental design [28]; and the remaining two studies used a single group before and after intervention study with no comparator [20,45]. Service provision for hypertension care appears to be a widely established intervention in community pharmacies and this service was similarly reported in three of the studies [20,28,45]. Among the twelve studies that focused on infectious diseases, only two adopted a quasi-experimental design [25,55], which sought to evaluate the effectiveness of intervention for malaria, pneumonia, and diarrhea, and another two studies employed a cluster-randomized trial [58] and randomized interventional study [54] to examine the impact of providing rapid diagnostic tests for malaria case detection and treatment. Similarly, from the eleven studies that focused on sexual and reproductive health, only one study was conducted using a controlled interventional design [23]. On the other hand, out of the five included studies focused on antimicrobial resistance, four studies were intervention evaluation studies [62,63,64,65]. Overall, the majority of the interventional studies included in this scoping review were a single group with non-controlled designs. In addition, there were also issues concerning the reporting of the details of the study design and providing further information on the processes involved for the randomized and controlled intervention studies, such as the randomization procedures and details of the control group. This indicated that the located studies were less rigorous in their design, and the agenda of pharmaceutical public health was not supported by strong evidence.

Similarly, the thirteen studies focused on NCDs utilized diverse techniques when assessing service provision, indicating a heterogeneity between the included studies in terms of how the studies were performed and the areas being investigated. In five of the studies, the service provision was confirmed by interviewing pharmacy professionals about their involvement in particular areas of concern [18,19,21,49,50], indicating a high likelihood of recall bias occurrence. In the remaining studies, a variety of services were delivered to different clients, and information was collected through participant interviews before and after service delivery, as well as through a simulated client visit [20,28,43,44,45,46,47,48]. Interestingly, in three of the hypertension intervention studies, pharmacy professionals provided three distinct services: blood pressure measurement, medication and lifestyle counseling using technology [45]; provision of a hypertension information diary for daily use [28]; and screening and referral of hypertension cases [20]. This implies that there is a wide margin for the interpretation of the data in the included studies due to the variety of services provided, and the studies frequently examined service feasibility and acceptability, making drawing conclusions regarding the effectiveness and sustainability problematic.

The findings of this review also highlighted the limitations across the range of public health interventions in the region. Many of the studies were feasibility and acceptability studies conducted in a narrow study area, as well as in a small number of LMICs in Africa, which resulted in little evidence of the service effectiveness, issues of broad generalizability of the findings, and sustainability. As a result, extrapolating the findings to a broader pharmacy environment, whether within the same country or across LMICs in Africa, is difficult. Evidence from Nigeria and South Africa [28,44,45], for example, indicated pharmacist-led service improvement in the areas of NCDs; however, this was not the case in Ethiopia [18]. This suggests that, while there is much to be learned from other countries when developing pharmacy public health programs, they must be reproduced and appraised in a variety of contexts in order to expand and strengthen the evidence base. Furthermore, nearly half of the included studies found limitations in the public health service success, which might indicate structural and institutional impediments to providing these services [18,19,21,23,24,47,48,49,50,53,60,61,65,66,67,69].

If pharmacy professionals in LMICs in Africa aspire to expand their role and contribute more effectively to the healthcare system, then resolving the barriers associated with service provision at all levels is critical. The approach must be multi-faceted and include both a willingness of pharmacy professionals to become more involved in the new roles and upgrade themselves, as well as developing policies that recognize and utilize the potential of pharmacy professionals to provide expanded services. To realize this, they should engage with policymakers, which is very important though the process of achieving this is not an easy task and requires sustained efforts over a period of years. In addition to policy change, attention must be directed to increasing the public awareness of both service provision and pharmacy professionals’ roles in public health. Lessons from the high-income countries show that public understanding is critical to enhancing the uptake of novel services [73,74,75]. When consumers are not aware of the breadth of the pharmacy professional’s role and expertise in the delivery of services other than dispensing medications, they will not seek those services in the pharmacy and will remain unaware. As a result, the services become underutilized. Therefore, addressing these barriers is very important to increase the implementation and utilization of the services.

### 4.1. Strengths and Limitations of this Scoping Review

To the authors’ knowledge, this is the first scoping review highlighting pharmacy professionals’ contributions in the areas of public health from the context of LMICs in Africa. This review provides an important resource for assessing pharmacy professionals’ current practice and reveals the variety of public health services reported in various countries. As a result, it provides valuable baseline information for various stakeholders, including researchers and practitioners, to develop an evidence-informed approach to identifying potential areas for further research and influencing policy and practice in the pharmacy profession.

The strength of this review is that it was conducted and reported using the standard guideline provided by the Preferred Reporting Items for Systematic Review and Meta-Analysis Extension for Scoping Reviews [35]. To locate the relevant evidence on pharmacy professional-delivered interventions, a comprehensive and systematic searching of the evidence was conducted. The searching involved a broad range of literature from six electronic databases (Medline, Embase, International Pharmaceutical Abstract, PsycInfo, Maternity and Infant Care Database, and Cochrane database of systematic review); hand searching of ten common journals that focus on health issues of the African continent; theses and dissertation related gray literature searching on EBSCO Open dissertations, Networked Digital Library of Theses and Dissertations (NDLTD) global Electronic Theses and Dissertation (ETD) search, and BIELEFELD Academic Search Engine (BASE); and the searching of libraries of the International Society for Pharmacoeconomics and Outcomes Research, WHO, FIP, Africa Centers for Disease Control and Prevention. The searching strategy was designed to be both sensitive and exhaustive in order to gain insight into the range and diversity of interventions that have come under investigation. In addition, the inclusion criteria of this study were broad, with no restrictions on the type of medicine retail outlets, qualification of professionals, study design, interventions, or health conditions indicating the low likelihood of missing relevant literature from inclusion. Furthermore, to ensure the quality and consistency of the screening, two-phase screening, first on the title and abstract and again on full-text screening, was conducted. Finally, the included articles were screened twice, considering their variation in terms of design, intervention, and setting.

One of the review’s limitations was that the findings were narratively described due to the wide range of literature covered in terms of service type, design, and population. This may also cause some degree of judgment about the findings to be emphasized. There was also a limitation of not having a full double screening approach; however, this was a scoping study with a systematic search approach, and throughout the process, a second author (DAO) was involved in identifying the search terms, agreeing the eligibility criteria, discussing and reviewing the identified studies, including random double screening and checks. Despite this review’s use of a broad eligibility criteria, there were restrictions on the language and year of publication; only studies reported in the English language and published after 2010 were included. Due to these filtering techniques, relevant articles might be excluded. Furthermore, in this review, the risk of bias for the included studies only conducted a grading of the strength of evidence, indicating that problems with the execution and design of the included studies may raise questions on the validity of the findings. It is, however, important to note that as a scoping review, the study has been used to successfully determine the extent, range, and nature of evidence/literature on a specific topic rather than establishing causality or evaluating the quality of the studies, risk of bias assessment was not appropriate. Scoping reviews are intended to identify as many studies on a specific topic as possible rather than critically evaluating the quality of individual studies. The scoping review identified and mapped the broad literature and summarized it based on the emerging themes. The identified studies were also highly heterogeneous in terms of design, service delivery, and settings, making it difficult to apply a single tool for assessment. A hierarchy of evidence approach was used to grade studies strength focusing on the study designs that were used.

### 4.2. Implication

This scoping review has important implications for future pharmacy research and practice. Currently, there is insufficient high-level published evidence of pharmacy professionals’ contribution to public health practice in Africa’s LMICs. Pharmacy professionals’ contributions/leadership within public health can potentially be termed under the umbrella Pharmaceutical Public Health, defined as “the application of pharmaceutical knowledge, skills and resources to the science and art of preventing disease, prolonging life, promoting, protecting and improving health for all through organized efforts of society” [76]. The included studies were conducted in a small number of LMICs in Africa, with several service success limitations. This makes the findings’ generalizability and service sustainability difficult. Future research is needed to improve the quality of the evidence, which will then serve as a foundation for rational health policy reform, allowing pharmacy professionals to make major contributions to public health efforts in the region. We recommend the following for future research considerations:

- Randomized and interventional studies that compare the effectiveness of the delivery public health services by pharmacy professionals.
- Studies exploring the experiences and challenges faced by pharmacy professionals in providing public health services and identifying strategies to overcome these barriers.
- Studies investigating the impact of policy and regulatory changes on the delivery of public health services by pharmacy professionals in LMICs.
- Studies evaluating the effectiveness of interprofessional collaboration between pharmacists and other health professionals in delivering public health services in LMICs.

## 5. Conclusions

This is the first scoping review to uncover a growing and diverse research literature aimed at providing evidence of the public health interventions delivered by pharmacy professionals in Africa’s LMICs. The identified public health interventions provided by pharmacy professionals in LMICs in Africa were categorized into five themes: NCDs, infectious diseases, sexual and reproductive health, antimicrobial resistance, and other health conditions. This highlights the attempts have been made over the past decade to expand the scope of pharmacy practice in the region through piloting the introduction of new services in both pharmacy and public health practice. However, the rate of such expansion has been relatively slow, and a strong evidence base was not available, due to a small number of studies across the region with limited RCTs, single arm before and after intervention, and cross-sectional studies with a high risk of introducing bias. Even though there advancement in delivering education and trainings for pharmacy professionals, the highlighted barriers that have limited the progress of service provision include a lack of health promotion activities in pharmacies, a lack of staff or resources, a lack of confidence, a lack of public recognition, a lack of collaboration with other health care professionals, and a lack of supportive policies. There are important opportunities available for pharmacy professionals to expand their practice into public health areas, mainly in the health improvement and protection domains, but this will require focused and well-planned efforts to overcome the challenges highlighted in this review. These efforts should center on a coordinated strategy to change public views, as well as the regulatory system, in order to achieve pharmacy professionals’ huge potential as a recognized resource for the delivery of public health services.

## Figures and Tables

**Table 1:** Summary of study characteristics by themes.

Themes	Included Studies and Aims of Interventions	Study Settings (*n*)	Intervention Component, Mode of Delivery and Training (*n*)	Study Designs (*n*)	Number of Settings
NCDs	15 studies identified Interventions: Cardiovascular risk factors including diabetes, hypertension, obesity	Nigeria (7) Ethiopia (4) Ghana, South Africa, Sudan and Tanzania (1 each) Community pharmacies (12), and health centers or hospitals (3)	Risk identification and reduction screening for cardiovascular risk factors (7) Disease management: interventions included counselling or advice, education, medication management (8) In person service delivery (15) Use of mobile health technology (1) Staff training on service delivery (7) Length of training reported in 3 studies and were one or two days	Cross-sectional study design to gather and analyze data on service provision (10) Single-blind randomized controlled trial (1) Quasi-experimental design (1) Single group before and after intervention studies (3)	More than one setting or multi-center (11) More than 100 individuals (6)
Infectious Diseases	12 studies identified: Malaria (8) Diarrheal disease (6) Pneumonia (5) Tuberculosis (1)	Tanzania (3) Ghana (2) Ethiopia (2) Uganda (2) Nigeria, Kenya and Sudan (1 each) Community pharmacies or accredited drug dispensing outlets or drug shops or licensed chemical shops or drug stores/vendors	Interventions focused on testing and treatment of conditions Additional interventions: referral to others, counselling and education In person service delivery (12) Staff training on service delivery (6) Length of training reported in 5 studies, and were 3 days (2), 5 days (2) and 1 week (1)	Cross-sectional study design (8) Mixed-method design including survey and qualitative interview (1) Quasi-experimental design (2) Cluster-randomized trial (1) Randomized interventional study (1)	More than one setting or multi-center (12) Data collected from 100 or more participants (7)
Sexual and reproductive health	11 studies were identified: sexually transmitted infections (STIs) (5), and reproductive health (6)	Kenya (5) Ethiopia (2) Uganda, Ghana, and Sudan (1 each), and Nigeria and Kenya (1) Community pharmacies or licensed chemical seller or drug shops	Interventions focused on screening and treatment of STIs, and family planning Additional interventions: referral to others, counselling and education In person service delivery (11) Staff training on service delivery (7) Length of training reported in one study, and was 6 weeks	Cross-sectional study design (10) Quantitative (8) Qualitative design involving FGDs, in-depth interviews, KIIs (2) Controlled interventional design (1)	More than one setting or multi-center (11) Data collected from 100 or more participants (4)
Antimicrobial resistance	5 studies were identified aimed at evaluating antibiotic stewardship interventions	South Africa (3) Ethiopia (1) Nigeria (1) Community pharmacies (1) Hospital (4)	Interventions: reviewing and auditing antimicrobial therapy, protocol development, feedback, and in-service education In person service delivery (5) Additional written protocol and mobile phone messages (2) Staff training on service delivery (2)	Cross-sectional (1) Single group before and after intervention (4)	More than one setting or multi-center (5) Data collected from 100 or more participants (4)
Other health conditions	5 studies focused on back pain, dyspepsia, immunization, oral health, unused medicine or waste, minor ailment, and community health risk identification	Ethiopia (3) Nigeria (1) Sudan (1) community pharmacies	Vaccine distribution, providing information, conducting need assessments, counselling, diagnosing and treating conditions, and referral to physician In person service delivery (5)	cross-sectional design (5)	More than one setting or multi-center (4) Data collected from 100 or more participants (1)

FGDs: Focus Group Discussions, KIIs: Key Informant Interviews.

## Data Availability

This article and its supplementary files contain all data generated or analysed during the conduct of this study.

## Supplementary Materials

The following supporting information can be downloaded at: https://www.mdpi.com/article/10.3390/pharmacy11010024/s1, File S1: Details of eligibility criteria; File S2: The completed Preferred Reporting Items for Systematic reviews and Meta-Analyses extension for Scoping Reviews (PRISMA-ScR) Reporting Checklist; File S3: Detailed search strategy; File S4: Full details of included studies. References [77,78,79,80] are cited in the Supplementary Materials.

## Abbreviations

BMI: Body Mass Index; FIP: International Pharmaceutical Federation; LMICs: Low- and -Middle-Income Countries; NCDs: Noncommunicable diseases; PRISMA-ScR: Preferred Reporting Items for Systematic Review and Meta-Analysis Extension for Scoping Reviews; RCT: Randomized Controlled Trial; SDGs: Sustainable Development Goals; STIs: Sexually Transmitted Infections; WHO: World Health Organisation.
