# Supplementary material for: Public Health Interventions Delivered by Pharmacy Professionals in Low- and Middle-Income Countries in Africa: A Systematic Scoping Review
Source: Pharmacy (Basel). 2023 Jan 30;11(1):24. doi: 10.3390/pharmacy11010024 (PMC9960443; doi:10.3390/pharmacy11010024)
Supplement: Supplementary file 1 [file pharmacy-11-00024-s001.zip › pharmacy-2158630-supplementary.pdf]

Supplementary files

Supplementary file S1: Details of eligibility criteria.

Supplementary file S2: The completed Preferred Reporting Items for Systematic reviews and Meta-Analyses extension for Scoping Reviews (PRISMA-ScR) Reporting Checklist.

Supplementary file S3: Detailed search strategy.

Supplementary file S4: Full details of included studies

Supplementary file S1

Details of eligibility criteria

**Inclusion criteria**	**Exclusion criteria**
**1.** Conducted between 2010 and 2020, as the literature has grown in recent years, and FIP has expanded the scope of pharmacy practice to incorporate public health since 2006 in collaboration with WHO (1).	**1.** Publication date: before 2010
**2.** Reported in the English language	**2.** Language, not English due to the costs and time required to translate studies.
**3.** Studies undertaken following either experimental (e.g. randomized and non-randomized controlled trials, before and after studies) or epidemiological designs (e.g. cross-sectional,	**3.** Country: Other than LMICs in Africa

cohort, or case-control studies). In addition, any existing literature such as organizational reports, theses, dissertations, conference papers, systematic or literature reviews, case reports, and commentaries.	
**4.** A literature of pharmacy professionals or medicine retail outlet public health interventions	**4.** Intervention: Not by pharmacy professional or medication retail outlets
**5.** A study conducted in LMICs in Africa<br>According to the World Bank country classifications based on income level, 53 African countries are classified as LMICs (2).<br>These are: Algeria, Angola, Benin, Botswana, Burkina Faso, Burundi, Cape Verde, Cameroon, Central African Republic, Chad, Comoros, Democratic Republic Congo, Congo, Cote d'Ivoire, Djibouti, Egypt, Equatorial Guinea, Eritrea, Eswatini, Ethiopia, Gabon, Gambia, Ghana, Guinea, Guinea-Bissau, Kenya, Lesotho, Liberia, Libya, Madagascar, Malawi, Mali, Mauritania, Mauritius, Morocco, Mozambique, Namibia, Niger, Nigeria, Rwanda, Sao Tome and	**5.** No mention of public health involvement or public health intervention in abstract<br>**6.** Training or research methods study, student training, or students' views of pharmacy<br>**7.** Focus: only medicine dispensing or prescription filling<br>**8.** Pharmacy student-led interventions as they are not licensed to provide the service.<br>**9.** Setting: Not medication retail outlet<br>**10.** Intervention not delivered by pharmacy professional or pharmacy team

Principe, Senegal, Sierra Leone, Somalia, South Africa, South Sudan, Sudan, Tanzania, Togo, Tunisia, Uganda, Zambia, and Zimbabwe	**11.** Interventions delivered by unlicensed or unregistered pharmacy professionals,
	**12.** Studies solely of views that are not set in the LMICs in Africa
	**13.** Studies of only knowledge and attitude about pharmacy public health service.
	**14.** Data collected before 2008
	**15.** Only needs assessment evidence
	**16.** Intervention prevalence only
	**17.** Scope is broader than pharmacy professional/medication retail outlet and pharmacy data not presented.
	**18.** Scope is out of health improvement, health protection public and health practice areas for pharmacy, and health service delivery and quality.
	**19.** Comparison of views from one country to another
	**20.** Duplicate
	**21.** Cannot be located

Supplementary file S2

Completed Preferred Reporting Items for Systematic reviews and Meta-Analyses extension for Scoping Reviews (PRISMA-ScR) Checklist

SECTION	ITEM	PRISMA-ScR CHECKLIST ITEM	REPORTED ON PAGE #
**TITLE**			
Title	1	Identify the report as a scoping review.	1
**ABSTRACT**			
Structured summary	2	Provide a structured summary that includes (as applicable): background, objectives, eligibility criteria, sources of evidence, charting methods, results, and conclusions that relate to the review questions and objectives.	2
**INTRODUCTION**			
Rationale	3	Describe the rationale for the review in the context of what is already known. Explain why the review questions/objectives lend themselves to a scoping review approach.	3-7
Objectives	4	Provide an explicit statement of the questions and objectives being addressed with reference to their key elements (e.g., population or participants, concepts, and context) or other relevant key elements used to conceptualize the review questions and/or objectives.	7
**METHODS**			
Protocol and registration	5	Indicate whether a review protocol exists; state if and where it can be accessed (e.g., a Web address); and if available, provide registration information, including the registration number.	8
Eligibility criteria	6	Specify characteristics of the sources of evidence used as eligibility criteria (e.g., years considered, language, and publication status), and provide a rationale.	9
Information sources*	7	Describe all information sources in the search (e.g., databases with dates of coverage and contact with authors to identify additional sources), as well as the date the most recent search was executed.	8,9
Search	8	Present the full electronic search strategy for at least 1 database, including any limits used, such that it could be repeated.	8,9
Selection of sources of evidence†	9	State the process for selecting sources of evidence (i.e., screening and eligibility) included in the scoping review.	9-11
Data charting process‡	10	Describe the methods of charting data from the included sources of evidence (e.g., calibrated forms or forms that have been tested by the team before their	12

SECTION	ITEM	PRISMA-ScR CHECKLIST ITEM	REPORTED ON PAGE #
		use, and whether data charting was done independently or in duplicate) and any processes for obtaining and confirming data from investigators.	
Data items	11	List and define all variables for which data were sought and any assumptions and simplifications made.	12
Critical appraisal of individual sources of evidence§	12	If done, provide a rationale for conducting a critical appraisal of included sources of evidence; describe the methods used and how this information was used in any data synthesis (if appropriate).	NA
Synthesis of results	13	Describe the methods of handling and summarizing the data that were charted.	12
**RESULTS**			
Selection of sources of evidence	14	Give numbers of sources of evidence screened, assessed for eligibility, and included in the review, with reasons for exclusions at each stage, ideally using a flow diagram.	11, 13
Characteristics of sources of evidence	15	For each source of evidence, present characteristics for which data were charted and provide the citations.	13-25
Critical appraisal within sources of evidence	16	If done, present data on critical appraisal of included sources of evidence (see item 12).	NA
Results of individual sources of evidence	17	For each included source of evidence, present the relevant data that were charted that relate to the review questions and objectives.	48
Synthesis of results	18	Summarize and/or present the charting results as they relate to the review questions and objectives.	13-25
**DISCUSSION**			
Summary of evidence	19	Summarize the main results (including an overview of concepts, themes, and types of evidence available), link to the review questions and objectives, and consider the relevance to key groups.	27-29
Limitations	20	Discuss the limitations of the scoping review process.	30
Conclusions	21	Provide a general interpretation of the results with respect to the review questions and objectives, as well as potential implications and/or next steps.	32
**FUNDING**			
Funding	22	Describe sources of funding for the included sources of evidence, as well as sources of funding for the scoping review. Describe the role of the funders of the scoping review.	1

Supplementary file S3

Search strategy

An extremely similar search strategy was followed on all electronic databases.

Database: Ovid MEDLINE(R) <1946 to August Week 3 2021>

Search Strategy:

----

1 Pharmacists/ (18675)

2 Pharmacy Technicians/ (806)

3 Druggist?.ab,kf,ti,tw. (116)

4 (communit$ adj pharmacist?).ab,kf,ti,tw. (2724)

5 Chemist?.ab,kf,ti,tw. (6193)

6 Pharmacies.ab,kf,ti,tw. (10345)

7 Pharmacies/ (8570)

8 Pharmacy Service, Hospital/ (12072)

9 Pharmacy/ (8673)

10 Community Pharmacy Services/ (5138)

11 (Communit$ adj Pharmac$).ab,kf,ti,tw. (6066)

12 (Hospital adj pharmac$).ab,kf,ti,tw. (3948)

13 (Drug$ adj stor$).ab,kf,ti,tw. (590)

14 (Rural adj drug$ adj vendor?).ab,kf,ti,tw. (4)

15 (Inpatient$ adj pharmac$).ab,kf,ti,tw. (163)

16 (Outpatient$ adj Pharmac$).ab,kf,ti,tw. (601)

17 (Emergenc$ adj Pharmac$).ab,kf,ti,tw. (38)

18 (Drug$ adj retail$ adj outlet?).ab,kf,ti,tw. (14)

19 (Medication? adj retail$ adj outlet?).ab,kf,ti,tw. (2)

20 1 or 2 or 3 or 4 or 5 or 6 or 7 or 8 or 9 or 10 or 11 or 12 or 13 or 14 or 15 or 16 or 17 or 18 or 19 (54400)

21 Health Promotion/ (77373)

22 (Health adj improvement$).ab,kf,ti,tw. (3190)

23 (Health adj well-being).ab,kf,ti,tw. (836)

24 life style/ or healthy lifestyle/ (61978)

25 body weight/ or body weight changes/ or overweight/ (217658)

26 (Weight adj management$).ab,kf,ti,tw. (5854)

27 Self Care/ (34450)

28 Advi#e.ab,kf,ti,tw. (53277)

29 Directive Counseling/ or Sex Counseling/ or Counseling/ or Distance Counseling/ (40788)

30 (Minor adj ailment$).ab,kf,ti,tw. (255)

31 Health Knowledge, Attitudes, Practice/ or Health Education/ (172535)

32 Campaign$.ab,kf,ti,tw. (40470)

33 (Public adj health adj campaign$).ab,kf,ti,tw. (1010)

34 "Early Detection of Cancer"/ or Mass Screening/ (131980)

35 (Cancer adj awareness).ab,kf,ti,tw. (1018)

36 smoking cessation/ or smoking reduction/ or "tobacco use cessation"/ (31477)

37 immunization/ or immunization, passive/ or immunization schedule/ or immunization, secondary/ or vaccination/ or mass vaccination/ (165232)

38 risk assessment/ or risk factors/ (1082776)

39 drinking behavior/ or alcohol drinking/ or health behavior/ (127165)

40 (Alcohol$ adj3 campaign$).ab,kf,ti,tw. (157)

41 Mental Health/ (46270)

42 (mental adj3 well-being).ab,kf,ti,tw. (4326)

43 exercise/ or physical conditioning, human/ or running/ or walking/ (174098)

44 (physical$ adj activ$).ab,kf,ti,tw. (111043)

45 body mass index/ (136211)

46 Harm Reduction/ (3542)

47 Dental Care/ (21933)

48 (Dental adj3 health).ab,kf,ti,tw. (11432)

49 (fall$ adj hip$ adj fracture$).ab,kf,ti,tw. (37)

50 Primary Health Care/ (83428)

51 (Health adj check$).ab,kf,ti,tw. (5529)

52 Sexual Behavior/ or Sexual Health/ (60691)

53 Long-Acting Reversible Contraception/ or Contraception, Barrier/ or Contraception/ or Contraception Behavior/ or Hormonal Contraception/ (25608)

54 health services accessibility/ or health equity/ or healthcare disparities/ (96122)

55 (outreach$ adj service$).ab,kf,ti,tw. (714)

56 "Diet, Food, and Nutrition"/ or Diet Therapy/ or Diet/ (178910)

57 Nutrition Therapy/ or Nutrition Assessment/ (18649)

58 (Disease$ adj Prevention$).ab,kf,ti,tw. (16471)

59 (Disease$ adj Screen$).ab,kf,ti,tw. (1734)

60 Diagnosis/ or Early Diagnosis/ (45832)

61 (Health adj protection$).ab,kf,ti,tw. (3521)

62 Antimicrobial Stewardship/ or Infection Control/ (29821)

63 Practice Guidelines as Topic/ (124931)

64 Evidence-Based Medicine/ (74789)

65 accidents/ or disease outbreaks/ or epidemics/ or emergencies/ or endemic diseases/ (165043)

66 hazardous waste/ or medical waste/ or dental waste/ or medical waste disposal/ (6062)

67 Adverse Drug Reaction Reporting Systems/ or "Drug-Related Side Effects and Adverse Reactions"/ or Pharmacovigilance/ (40826)

68 (medicine$ adj recall$).ab,kf,ti,tw. (5)

69 risk assessment/ or safety management/ (305604)

70 (safety adj alert$).ab,kf,ti,tw. (387)

71 "Referral and Consultation"/ (70285)

72 Oral Hygiene/ or Hand Hygiene/ or Hygiene/ (31611)

73 global health/ or public health/ (134846)

74 (Healthcare adj3 (public adj health)).ab,kf,kw,tw. (603)

75 (Medicine$ adj3 review$).ab,kf,kw,tw. (3942)

76 (Drug$ adj3 evaluation$).ab,kf,kw,tw. (8990)

77 program evaluation/ or clinical audit/ or medical audit/ or quality improvement/ or "utilization review"/ (118470)

78 guideline adherence/ or "outcome and process assessment, health care"/ (61352)

79 drug misuse/ or medication errors/ or medication reconciliation/ (14858)

80 diabetes mellitus/ or diabetes mellitus, type 1/ or diabetes mellitus, type 2/ or diabetes, gestational/ or diabetic ketoacidosis/ (339842)

81 hypertension/ or essential hypertension/ or hypertension, malignant/ or hypertension, pregnancy-induced/ (248284)

82 cardiovascular diseases/ or cardiovascular abnormalities/ or cardiovascular infections/ or heart diseases/ or pregnancy complications, cardiovascular/ or vascular diseases/ (284408)

83 asthma/ or asthma, aspirin-induced/ or asthma, exercise-induced/ or asthma, occupational/ or asthma-chronic obstructive pulmonary disease overlap syndrome/ or status asthmaticus/ (133128)

84 pulmonary disease, chronic obstructive/ or asthma-chronic obstructive pulmonary disease overlap syndrome/ or bronchitis, chronic/ or pulmonary emphysema/ (60038)

85 21 or 22 or 23 or 24 or 25 or 26 or 27 or 28 or 29 or 30 or 31 or 32 or 33 or 34 or 35 or 36 or 37 or 38 or 39 or 40 or 41 or 42 or 43 or 44 or 45 or 46 or 47 or 48 or 49 or 50 or 51 or 52 or 53 or 54 or 55 or 56 or 57 or 58 or 59 or 60 or 61 or 62 or 63 or 64 or 65 or 66 or 67 or 68 or 69 or 70 or 71 or 72 or 73 or 74 or 75 or 76 or 77 or 78 or 79 or 80 or 81 or 82 or 83 or 84 (3991602)

86 Angola/ (1068)

87 Benin/ (1712)

88 Botswana/ (1953)

89 Burkina Faso/ (3490)

90 Burundi/ (670)

91 Cape Verde/ (230)

92 Cameroon/ (5887)

93 Central African Republic/ (801)

94 Chad/ (755)

95 Comoros/ (333)

96 Democratic Republic Congo/ (0)

97 Congo/ (1932)

98 Cote d'Ivoire/ (3281)

99 Djibouti/ (241)

100 Egypt/ (15911)

101 Equatorial Guinea/ (283)

102 Eritrea/ (380)

103 Eswatini/ (653)

104 Ethiopia/ (14873)

105 Gabon/ (1527)

106 Gambia/ (2517)

107 Ghana/ (9300)

108 Guinea/ (1158)

109 Guinea-Bissau/ (984)

110 Kenya/ (17398)

111 Lesotho/ (464)

112 Liberia/ (1262)

113 Libya.ab,hw,kw,ti. (1517)

114 Madagascar/ (3648)

115 Malawi/ (5893)

116 Mali/ (2493)

117 Mauritania/ (468)

118 Mauritius/ (589)

119 Morocco/ (6083)

120 Mozambique/ (2676)

121 Namibia/ (1175)

122 Niger/ (1270)

123 Nigeria/ (30575)

124 Rwanda/ (2721)

125 "Sao Tome and Principe"/ (24)

126 Senegal/ (5934)

127 Sierra Leone/ (1698)

128 Somalia/ (1704)

129 South Africa/ (44995)

130 South Sudan/ (191)

131 Sudan/ (4903)

132 Tanzania/ (12327)

133 Tunisia/ (8735)

134 Uganda/ (13312)

135 Zambia/ (4900)

136 Zimbabwe/ (6117)

137 Africa/ (31362)

138 Togo/ (1198)

139 Algeria/ (3258)

140 86 or 87 or 88 or 89 or 90 or 91 or 92 or 93 or 94 or 95 or 96 or 97 or 98 or 99 or 100 or 101 or 102 or 103 or 104 or 105 or 106 or 107 or 108 or 109 or 110 or 111 or 112 or 113 or 114 or 115 or 116 or 117 or 118 or 119 or 120 or 121 or 122 or 123 or 124 or 125 or 126 or 127 or 128 or 129 or 130 or 131 or 132 or 133 or 134 or 135 or 136 or 137 or 138 or 139 (270140)

141 20 and 85 and 140 (611)

142 limit 141 to (english language and yr="2010 - 2020") (347)***************************

To optimize the search in terms of the sensitivity and specificity of the overall search strategy results, the database's-controlled vocabulary, Medical Subject Heading (MeSH) term focusing on the scope was used. Besides, searching using free-text terms (natural vocabulary) in conjunction with relevant database thesaurus terms was also used to yield the best results. Applying truncation, wildcard, and/or proximity (TWP) operators specific to the Ovid platform for the six databases were applied on the free text terms to increase the quality of the retrieval of the intended results. The keywords and terms in each category were then combined with the Boolean operator "OR" to the MeSH terms identified from the database. Each was then added to the main search using the Boolean operator "AND".

Hand-searching of key journals

List of ten common journals include: the Africa Health Journal, West African Journal of Pharmacy, the Journal of Public Health in Africa, the African Journal of Diabetes Medicine, Tropical Journal of Pharmaceutical Research, Ethiopian Pharmaceuticals Journal, African Journal of Reproductive Health, African Health Sciences, African Journal of Primary Health Care & Family Medicine, and Ethiopian Medical Journal.

Supplementary file S4

Full details on types of studies included, aims of interventions, study settings and design, intervention component, mode of delivery and training

Noncommunicable diseases (NCDs)

This section focuses on pharmacy professionals' service provision for NCDs public health intervention theme. NCDs, often known as chronic diseases, are long-term conditions caused by a combination of genetic, physiological, environmental, and behavioral factors (3).

Included studies and aims of the interventions

Fifteen studies were identified that investigated the service provision of pharmacy professionals in NCDs prevention and control (4-18). The interventions primarily targeted cardiovascular disease risk factors such as diabetes, hypertension, obesity and weight management, dyslipidemia, physical inactivity, an unhealthy diet, smoking, and alcohol abuse. In eight studies, service delivery simultaneously targeted at least two risk factors for cardiovascular disease (5, 7, 9, 11-15). In three other studies, service delivery was solely focused on hypertension (6, 8, 10). In one study, four different health conditions were targeted at the same time, including hypertension, insomnia, osteoarthritis, and anemia (4). The service delivery in the remaining three studies focused on diabetes (16), asthma (18), and anemia in pregnancy(17). Almost all of the studies investigated how pharmacy professionals could broaden their service provision beyond dispensing to include more involvement in public health issues. They were primarily intended to assess the effectiveness of the service at the level of the medicine retail outlet (4-8, 10, 11) or the level of involvement of pharmacy professionals in the provision of NCD-related services (9, 12-18). The service provision in seven studies was aimed at identifying risk factors for cardiovascular diseases (4, 7, 10-14). The service provision in the other six studies was aimed at treating disease conditions (5, 6, 8, 9, 15, 16). The

remaining two studies aimed to reduce anemia prevalence among pregnant women (17) and asthma control (18).

Study setting

Eleven of the fifteen studies were conducted in Nigeria (n=7)(4-7, 9, 17, 18) and Ethiopia (n=4)(13-16). The four remaining studies were conducted in Ghana (11), South Africa (8), Sudan (12), and Tanzania (10). In twelve studies, the service was provided in a community pharmacy (4-7, 9-16), whereas in three studies focusing on hypertension, asthma, and pregnant women, the service was provided at a health center or tertiary hospital (8, 17, 18). The majority of the studies (n=12) were multi-center studies that were carried out in more than one setting (5, 6, 8-16, 18). Only three studies on anemia, osteoarthritis, insomnia, and CVD risk factors were conducted in a single setting, either a health center or a tertiary hospital (4, 7, 17).

Intervention component, mode of delivery, and training

The NCDs theme's service delivery was primarily centered on detecting and lowering cardiovascular risk factors, as well as disease state management. Seven studies focused on risk identification and reduction offered screening for cardiovascular disease risk factors such as obesity, diabetes, hypertension, hypercholesterolemia, smoking, alcohol use, poor diet, and physical inactivity (5, 7, 10-14). Five of these studies recorded additional service provision, such as referral to other health professionals, health education and advice on smoking cessation, healthy diet, exercise, obesity, and weight management, the importance of blood cholesterol, blood pressure, and glucose control, and follow-up monitoring (5, 10-13). In the remaining eight studies, service provision was centered on disease management, with intervention components mostly consisting of counseling or advice, education, and medication management (4, 6, 8, 9, 15-18). The pharmacy professionals engaged offered advice and educational interventions on routine blood pressure, blood glucose, body mass index or waist circumference, and lipid profile

measurements, which assisted clients in setting goals and effectively manage their conditions. Furthermore, lifestyle counseling on the importance of physical exercise, salt restriction, vegetable consumption, weight loss, and smoking cessation was offered. In one study focused solely on diabetes management in Ethiopia, pharmacy professionals provided diabetic care in the form of advice or counseling, emphasizing the importance of regular exercise, weight control, diet, hypoglycemia detection and management, insulin administration, handling, and storage, the impact of over-the-counter and herbal drugs, proper foot and eye care techniques, regular screening for nephropathy, retinopathy, and neuropathy, and smoking cessation (16). In another study focused on anemia management among pregnant women in Nigeria, pharmacy professionals provided health education emphasizing four main points: adherence to routine medicines and planned prenatal checkups, a balanced diet, personal and environmental hygiene, and the significance of utilizing insecticide-treated nets (17).

In all of the studies, the service delivery to the clients was done in person (4-18). In one Nigerian study, service delivery was aided by the use of Mobile Health (mHealth) technology (6). In another South African study, patients were given a hypertension information diary to utilize on a daily basis, as well as 15–30 minutes of patient counseling and education about hypertension and its treatment, as well as how to use the diary properly(8). Seven studies reported on staff training for service delivery (5, 6, 8, 10, 11, 17, 18). Three studies recorded the length of the training, with one giving a one-day training (5) and the other two offering a two-day training (11, 17).

Study design

A combination of study designs was used to investigate service provision by pharmacy professionals in the NCDs theme. Ten studies used a cross-sectional study design to gather and analyze data on service provision(4, 5, 7, 9, 10, 12-16). One study utilized a single-blind randomized controlled trial (18), another study used a quasi-experimental

design (8), and the remaining three studies used a single group before and after intervention study without a comparator (6, 11, 17). In eleven of the studies (5, 6, 8-16), data were collected in more than one setting or multi-center, and in six studies, data were collected from a hundred or more individuals (4-7, 10, 11).

Infectious diseases

This section focuses on pharmacy professionals' service provision for infectious disease public health intervention theme. Communicable, or infectious diseases, are caused by microorganisms such as bacteria, viruses, parasites, and fungi that can be spread, directly or indirectly, from one person to another. Some are transmitted by insect bites, while others are caused by consuming contaminated food or drink (19). For this scoping review, sexually transmitted infections including gonorrhea, chlamydia, and human immunodeficiency virus infection, and acquired immunodeficiency syndrome (HIV/AIDS) were included under the sexual and reproductive health theme.

Included studies and aims of the interventions

A total of 12 studies were located in the infectious disease health topic (20-31). Eight of them were focused on the health condition of malaria, six were focused on diarrheal disease, another five studies were focused on pneumonia and one study was focused on tuberculosis. From the five studies examining service provision in more than one health condition, three of them were reported service provision for the three health conditions (malaria, pneumonia, and diarrhea) together, and the remaining two studies were reported for pneumonia and diarrhea together.

All of the studies were investigated the service expansion into medicine retail outlets (20-31). Eight of the studies focusing on malaria were aimed at investigating malaria testing and treatment (20-27). One study was additionally aimed at investigating preventive services (20). In two studies the services were offered for the rural population (26, 27), in four studies for children under the age of five years (21-24), and in the remaining two

studies the services were offered for the general population (20, 25). Of the studies focusing on pneumonia infection and diarrheal diseases, five studies were aimed at testing and treatment of the illnesses (22-24, 28, 29), and one study was only aimed at treating the illnesses (30). One study additionally offered referral services to other health professionals for further checkups (29). In four of the studies the service provision targeted children under the age of five years (22-24, 30) and in the remaining two studies the services were offered to the general population (28, 29). The primary aim of a study on tuberculosis was to improve tuberculosis case detection and referral via accredited drug dispensing outlets, and pharmacies (31).

Study setting

The included twelve studies focused on infectious disease health topic were conducted in seven countries such as Tanzania (n=3)(21, 23, 31), Ghana (n=2)(26, 27), Ethiopia (n=2)(29, 30), Uganda (n=2)(22, 24), Nigeria (n=1)(20), Kenya (n=1)(25) and Sudan (n=1)(28). All of the studies were conducted in community drug retail outlets (either in community pharmacies or accredited drug dispensing outlets, or drug shops, or licensed chemical shops, or drug stores/vendors). Similarly, all of them were conducted in more than one setting or multi-center studies to maximize service outreach.

Intervention component, mode of delivery, and training

The intervention components in the infectious disease health topic mainly focused on testing and treatment of the conditions. Additional intervention components such as referral to other professionals, advice or counseling, and prevention-focused education were also among the reported. In six out of eight malarial studies, the disease was detected by using the rapid diagnostic testing (RDT) technique (21, 22, 24-27). In two of these studies, the diagnosis was additionally guided by asking for a history of fever and microscopic examination (24, 25). In the remaining two studies malaria was detected by asking for the sign and symptoms of the disease such as fever and malaise (20, 23). In all

of the studies focused on diarrhea and pneumonia, the clinical sign and symptoms were used to detect the diseases (22-24, 28-30). In two of the studies focused on children under the age of five years, a respiratory timer was additionally used to detect pneumonia (22, 24). For the tuberculosis study, the disease was detected by assessing for persistent cough of two weeks duration, coughing blood, fever of two or more weeks duration, loss of weight, excessive night sweats, chest pains, shortness of breath, fatigue, and malaise (31).

Concerning the intervention components focused on treating the conditions, pharmacy professionals delivered artemisinin-based combination therapy to treat malaria in all of the malarial studies (20-27). A further advice or counseling service was provided in two studies (25, 27), and malaria prevention interventions such as promoting the use of insecticide-treated nets, and use of prophylactic drugs against malaria were reported in another study (20). To treat diarrheal disease pharmacy professionals delivered oral rehydration salts (ORS) with Zinc (22-24, 30), antimicrobials (30), over-the-counter medications (28, 29), and instruction on fluid and food intake (30). On the other hand, to treat pneumonia amoxicillin (22, 24) and over-the-counter medications with non-pharmacological interventions (28, 29) were delivered. In two pneumonia and diarrhea studies and one tuberculosis study pharmacy professionals referred the patients to other professionals for further investigation and treatment. In one study the management intervention for pneumonia did not report (23). In all of the studies, the service provision to the patients was carried out through a face-to-face encounter. Staff training for the service provision was reported in six studies (21, 22, 24-27). Five studies reported the duration of the training, with two offering a 3 days training (25, 26), the other two offering 2 days and 5 days training (21, 24), and another one reporting a one-week training (27).

Study design

Different study designs were utilized to investigate the service provision by pharmacy professionals in the infectious disease health topic. Eight studies used a cross-sectional

study design to collect and analyze the service provision data (20, 23, 25, 27-31). In one of these studies, a mixed-method design was employed involving survey and qualitative interview (27). Of the remaining studies, two adopted a quasi-experimental design (22, 24) which sought to evaluate the effectiveness of intervention for malaria, pneumonia, and diarrhea in drug shops, another two studies utilized a cluster-randomized trial (26) and randomized interventional study (21) to examine the impact of providing rapid diagnostic tests for malaria case detection and treatment in drug retailers. In all of the studies, the data was collected in more than one setting or multi-center, and in seven studies the data was collected from a hundred and more participants (21-27).

Sexual and reproductive health

This section focuses on the service provision of pharmacy professionals for the public health intervention theme of sexual and reproductive health. Sexual and reproductive health comprises providing services for family planning, the elimination of unsafe abortion, the prevention and control of sexually transmitted diseases, and the promotion of sexual health (32).

Included studies and aims of the interventions

A total of eleven studies were located on this health topic (12-14, 33-40). The studies were focused on testing, treatment, and prevention of sexually transmitted infections, and reproductive health. The primary aim of service provision via medicine retail outlets was to facilitate HIV/AIDS screening (40), to improve access to sexual health information and services such as HIV/AIDS prevention and awareness (12), sexually transmitted infections screening, and treatment services to the general population (13, 14, 37, 38), and to increase access to and reported use of contraceptive methods by women and youth population (12-14, 33-38).

Study setting

The majority of included studies examined sexual and reproductive health service provision in two countries, Kenya (n=5)(33, 37-40), and Ethiopia (n=2)(13, 14). The remaining four studies were examined the service provision in Uganda (34), Ghana (36), and Sudan (12) with one study in each, and one other study was conducted both in Nigeria and Kenya (35). All of the studies were conducted in community drug retail outlets (either in community pharmacies or licensed chemical seller shops, or drug shops). Similarly, all of them were conducted in more than one setting or multi-center studies to maximize service outreach.

Intervention component, mode of delivery, and training

The intervention components of studies focused on sexually transmitted infections (STIs) include screening for STIs by asking for the nature and duration of symptoms, recent sexual exposure, previous care-seeking for current illness, and providing treatment (39); advice on getting an HIV/AIDS test and getting partner treated (13, 39); referral service for sexually transmitted infection testing and counseling (37, 40); and education on HIV/AIDS awareness and prevention (12). In one study fliers were delivered to educate that emergency contraceptives do not protect against sexually transmitted infections including HIV/AIDS (37).

The intervention components of family planning studies include provision of contraceptive methods such as oral contraceptive pills (34), emergency contraceptives (33), injectables (34, 35, 38), implants (34), and condoms (33, 34) to clients. In one stud further fliers and brochures that describe emergency contraceptives are not 100% effective, and their indications (37). The other intervention component was provision of counseling on injectables and implants (34), and other contraceptive methods (12-14, 37). Associated with this, specific information on emergency contraceptive use such as the time frame within which they are effective, how they work, and their side effects were

also provided (13, 37). Apart from this, clients were referred to health facilities for screening, counseling, and injection (36). In all of the studies, the interventions were delivered to clients through a face-to-face encounter. For seven studies staff training for service provision was indicated (14, 34, 36-40), however, only one study has reported the duration of the training, six weeks training (34).

Study design

Different study designs were utilized to examine pharmacy professionals' service provision in sexual and reproductive health topics. Eight studies collected and analyzed quantitative cross-sectional data (12-14, 34-36, 39, 40). In two studies a qualitative design with a mixed method of data collection was employed (33, 38). These include focus group discussions, in-depth interviews, key informant interviews, and mystery shoppers. Only in one study, a controlled interventional design was employed (37), and this study involved intervention and control groups, with baseline and final assessments of emergency contraceptive provision by using mystery clients. In all of the studies, the data was collected in more than one setting or multi-center, and in four studies the data was collected from a hundred and more participants (34-36, 40).

Antimicrobial resistance

This section focuses on the service provision of pharmacy professionals for the public health intervention theme of antimicrobial resistance. Antimicrobials are medicines used to prevent and treat infections in humans, animals, and plants. These include antibiotics, antivirals, antiparasitic, and antifungals drugs (41). Antimicrobial resistance occurs when bacteria, viruses, fungi, and parasites change over time and no longer respond to these medicines (41).

Included studies and aims of the interventions

A total of five studies on antimicrobial resistance were included in this scoping review (13, 42-45). Four studies were intervention evaluations aimed at evaluating the impact of

pharmacy professional-driven antibiotic stewardship interventions to improve the appropriate use of antimicrobial agents (42-45). The remaining one study explored community pharmacy professionals' level of involvement and experiences on antimicrobial stewardship programs, rather than a specific intervention and is not further described (13).

Study setting

The included five studies were conducted in three countries, South Africa (n=3)(42-44), Ethiopia (n=1)(13), and Nigeria (n=1)(45). Four of the intervention evaluation studies were set in a hospital setting, and pharmacy professionals were responsible to deliver the interventions (42-45). All five of the studies were conducted in more than one hospital setting or multi-center studies.

Intervention component, mode of delivery, and training

The intervention components in two of the intervention evaluation studies were reviewing and auditing of antimicrobial therapy for basic process measures such as inappropriate duplicate antimicrobial coverage (42, 43), no cultures were obtained before antimicrobial therapy (42, 43), duration of antimicrobial therapy for greater than 7 or 14 days (42, 43), patients on four or more antimicrobial agents (42), and hang time compliance (43). Associated with hang time compliance pharmacy professionals provided in-service education to the nursing staff and physicians, and hung educational posters in nursing units to increase awareness of hang-time (43). In one study a pharmacist-driven, prospective audit and feedback service were delivered to facilitate adherence to perioperative antibiotic prophylaxis guidelines (44). In one another study pharmacy professionals developed and disseminated a department-level protocol for surgical antibiotic prophylaxis, delivered education for obstetricians and gynecologists, audited and provided feedback in the form of wall-mounted posters(45). In three of the studies, the interventions were delivered through a face-to-face encounter only (13, 42,

43). In the remaining two studies pharmacy professionals delivered the intervention through face-to-face encounters and additional written protocol (44, 45) or mobile phone messages (44). In two studies staff training for the intervention delivery was reported, however, the duration of the training was not stated (42, 44).

Study design

Four of the intervention evaluation studies were a single group before and after intervention studies (42-45). The remaining one study was a quantitative cross-sectional study that examined the involvement of pharmacy professionals to tackle antimicrobial resistance (13). In all of the studies, the data was collected in more than one setting or multi-center, and in four of the intervention evaluation studies, the data was collected from a hundred and more participants (42-45).

Other health conditions

This section provided a descriptive overview of studies that focused on the provision of services by pharmacy professionals to various health conditions that were not covered by the previous four public health intervention domains. These include dyspepsia, oral health, pain management, immunization, unused medicine or waste management, and identifying community health risks.

Included studies and aims of the interventions

This theme included a total of five studies that focused on various health conditions that were not covered by the previous four public health intervention themes (4, 12-14, 29). With one study for each health condition, the service provision was focused on back pain (29), dyspepsia (4), and community health risk identification (13). One of the remaining two studies focused on immunization and pain management (14), and the other on oral health and unused medicine or waste (12). Service provision in four studies aimed to improve community health by providing immunization, oral health education, minor ailment management, and information on unused or waste medicines, as well as

identifying community health risks (12-14, 29). The remaining study's service provision aimed to improve access to screening and treatment services for the rural population (4).

Study setting

The included five studies were conducted in Ethiopia (n=3) (13, 14, 29), Nigeria (n=1) (4), and Sudan (n=1) (12). All of the studies were conducted in a community pharmacy setting where pharmacy professionals were in charge of providing the services (4, 12-14, 29). Four of the studies involved multiple community pharmacies and were multicenter studies (12-14, 29). The remaining study, on the other hand, was conducted in a single community pharmacy (4).

Intervention component, mode of delivery, and training

The intervention components in three studies focused on improving the general community health were distributing a vaccine and treating pain (14), providing information on oral health and unused or waste medicines (12), and conducting need assessments to identify health risks (13). The intervention component of a study aimed at improving service accessibility for the rural population was dyspepsia management counseling (4). In the remaining study, the service provision involved back pain diagnosis based on patient history or by asking for signs and symptoms, medication and counseling provision, and referral to a physician (29). The services were delivered in all of the included studies through a face-to-face encounter (4, 12-14, 29).

Study design

A cross-sectional design was used for all of the studies to collect and analyze quantitative data. The services were provided in multiple community pharmacy settings in four of the studies (12-14, 29). The data was collected from a hundred or more participants in the remaining study, which was conducted in a single community pharmacy (4).

Summary of included studies in alphabetical order of the countries

Author(s)/year	Country	Aim of study	Health conditions	Publication/study type	Design	Summary of findings	Level of evidence
Ayele A. et al. 2018	Ethiopia	to document the involvement of community pharmacy professionals in the management of minor ailments and perceived barriers that limit their provision of such services.	Pain, Diarrhoea, Pneumonia	Descriptive	Mixed method study: simulated client visits and qualitative interview	In 61 (92.4%) cases community pharmacy professionals were enquired symptoms of low back pain, diarrhoea, and upper respiratory tract infections, and provided one or more medications. Extra non-pharmacological advice was provided for 28(45.9%) cases. In 26(42.6%) cases they advised to visit physician.	Level D
Tesfaye Z. et al. 2020	Ethiopia	To assess community's extent of use and approval of extended pharmacy services at community pharmacies.	Pain, Family planning, Weight management, Diabetes, Hypertension, Dyslipidemia, Immunization	Descriptive	Cross sectional survey	Community pharmacists provided extended pharmacy services in the areas of pain management, acute symptom management, family planning/sexual health, anthropometric measurements, immunization, minor ailments management, and health screening services for 72 clients.	Level D
Teka N. et al. 2018	Ethiopia	To assess the counselling practice of community pharmacists for diabetes mellitus patients	Diabetes	Descriptive	Cross sectional survey	Out of 300 community pharmacy professionals, half of them delivered appropriate counselling service on the appropriate time to administer each oral anti-diabetic drug and missed oral dose. Only 15.3% of the	Level D

						community pharmacy professionals gave proper counselling on the importance of continuous screening for nephropathy, retinopathy, and neuropathy.	
Belachew S. et al. 2020	Ethiopia	To assess community pharmacy professionals' opinions about metabolic syndrome, describe their perception level towards the effectiveness of the main interventions and explore their extent of involvement.	Weight management, Nutrition, Physical activity, Hypertension, Dyslipidemia, Smoking	Descriptive	Cross sectional survey	Community pharmacists were reported to have involved in counselling of weight loss, physical activity, salt restriction for hypertensives, cholesterol lowering diet, consumption of soluble fibres and vegetables, and smoking cessation.	Level D
Mengistu G. et al. 2019	Ethiopia	To assess self-reported knowledge and actual practices of community pharmacy professionals toward the management of diarrhoea.	Diarrhoea	Descriptive	Cross sectional survey and simulated patient visit	The result from survey revealed that more than 90% of the participants reported to recommend oral rehydration salt (ORS) plus zinc, and food intake for diarrhoea management. Majority of the community pharmacy professionals, 85%, also dispensed antimicrobial agents for the simulated patients.	Level D
Erku D et al. 2019	Ethiopia	To document the level of involvement of community pharmacy	Smoking, Physical-activity, Nutrition, Weight-	Descriptive	Cross sectional survey	Community pharmacy professionals were reported to have involved in counselling of	Level D

		professionals in the provision of public health services and the barriers to such involvement.	management, Alcohol use, Hypertension, Diabetes, Dyslipidemia, Antimicrobial resistance STIs, Family planning, Community health risks			smoking cessation, physical activity, healthy eating, weight management, alcohol consumption, contraceptives and STIs; screening of hypertension, diabetes, and dyslipidaemia; promoting antimicrobial stewardship programs; conducting needs assessments to identify health risks.	
Lebetkin E. et al. 2014	Ghana	To determine the feasibility and acceptability of shops' selling of injectables from the perspective of shop operators and their clients, and to examine whether the sale of the injectable in shops was associated with increased access to and reported use of the method.	Family planning	Descriptive	Cross sectional survey	Ninety-one (91.2%) shop operators reported provision of injectables, and almost all shop operators 93 (99%) referred clients to a hospital or health facility for injection. Two hundred and ninety-eight clients purchased injectables from the shops. Almost all clients 289 (97%) clients reported getting their injection at the health facility to which they were referred by the shop operators. Clients cited trust, convenience and commodities as key reasons for purchasing from a shop.	Level D
Marfo A. et al. 2016	Ghana	To explore the feasibility and acceptability of a pharmacist-	Hypertension	Interventional	Before/after study with no control group	Out of 170 participants agreed to be screened, 43 (25%) were pre-hypertensive, 42 (25%) had stage 1	Level C

		led hypertension preventative service in the community pharmacy				hypertension and 13 (8%) had stage 2 hypertension. The most frequent modifiable risk factors identified were lack of exercise 107 (63%), poor diet (42%) and obesity (21%). Lifestyle changes reported at 6 months by participants with pre-hypertension were weight reduction and reduced alcohol intake. Of the 34 participants who were referred to the physician, 10 (29%) were diagnosed with hypertension and an antihypertensive was prescribed.	
Ansah E. et al. 2015	Ghana	To examine the impact of providing rapid diagnostic tests for malaria in private drug retail shops.	Malaria	Interventional	Cluster randomize d controlled trial	Of 4603 clients, 3424 (74.4%) clients tested negative. The proportion of slide-negative clients who received any antimalarial was 590/1854 (32%) in the intervention arm and 1378/1570 (88%) in the control arm, P<0.0001. Of those who were slide-positive, 690/787 (87.8%) in the intervention arm and 347/392 (88.5%) in the control arm received an artemisinin combination therapy.	Level B
Kwarteng A. et al. 2019	Ghana	To assess the accuracy and perception of test-based management	Malaria	Descriptive	Mixed method study: survey and qualitative	In total, 947out of 1,426 febrile clients had a positive rapid diagnostic test	Level D

		of malaria using malaria rapid diagnostic test kits at private licensed chemical shops.			focus group discussions, and in-depth	(RDT) result. Of 947 RDT positive clients, 815 with uncomplicated malaria were treated with artemisinin-based combination therapy.	
Mugo P. et al. 2013	Kenya	To assess sexually transmitted infections (STIs) treatment and HIV testing referral practices among health providers.	STIs	Descriptive	Cross sectional survey and simulated visits	In both simulated visits and interviews pharmacists screened STIs. During simulated visits, 10% of pharmacies prescribed recommended antibiotics at recommended dosages and durations and, during interviews, 28% of pharmacies. HIV testing was recommended during 10% of simulated visits, and 20% of pharmacy interviews.	Level D
Mugo P. et al. 2015	Kenya	To assess the success of pharmacy referrals and uptake of HIV testing by young adult clients.	HIV/AIDS	Descriptive	Cross sectional survey	Out of 1490 pharmacy clients met targeting criteria, 1074 (72.1%) accepted a referral coupon, 377 (25%) reported for screening at study clinics, and 353 (24%) were HIV-1 tested.	Level D
Poyer S. et al. 2018	Kenya	To improve access to quality-assured RDTs through increasing availability, increasing demand for diagnostic testing, and improving the quality of	Malaria	Descriptive	Cross sectional survey and mystery client visits	In registered pharmacies, testing by RDT was 52.1% in 2014 and 56.3% in 2015. At least 75% of test positive patients received artemisinin-based combination therapy in both rounds, with no significant difference	Level D

		private sector fever case management.				between outlet types in either round. mystery clients received the correct negative diagnosis and did not receive an anti-malarial in 78.4% of visits to registered pharmacies.	
Gonsalves L. et al. 2019	Kenya	To assess private pharmacies as an existing source of injectable contraception for young Kenyans, and investigated the perceived quality of service provision.	Family planning	Descriptive	Mixed method study: mystery client visits and qualitative interviews, focus group discussions, in-depth and key informant interviews	Participants confirmed that injectable contraceptives were routinely delivered and administered in pharmacies during the qualitative interview and discussions. The mystery client visits revealed that injectables were delivered in 29/45 (65%) visits. In 44% of the visited pharmacies, the clients purchased and received the injection on site. In 12 visits clients were referred either to individual or clinic qualified to provide the injections.	Level D
Liambila W. et al. 2010	Kenya	To evaluate the provision of reproductive health information and services to users of emergency contraceptives by private pharmacists.	Family planning, STIs	Interventional	Before/after study with control group	The intervention and control group pharmacists provided emergency contraceptive (EC) pills for (87.8%), (86.2%), additional information on EC for (54.1%), (46.0%), offered regular family (FP) planning services for (11.2%), (9.2%), talked about STIs/HIV for (5.1%), (5.8%),	Level C

						offered regular FP and/or HIV/STI services for (12.2%), (11.5) clients respectively.	
Gonsalves L. et al. 2020	Kenya	To determine whether pharmacies in Coastal Kenya might be an untapped potential source of youth-friendly modern contraceptive sources.	Family planning	Descriptive	Mixed-method: qualitative design involving focus group discussions, in-depth interviews, key informant interviews and mystery client visit	Pharmacies were successfully provided emergency contraceptive pills and condoms to young people, and, they were satisfied with the quick, transactional interaction with pharmacy personnel.	Level D
Corroon M. et al. 2016	Kenya and Nigeria	To explore the role that drug shops and pharmacies play in the provision of contraceptive methods and factors associated with women's choice of where to obtain these methods.	Family planning	Descriptive	Cross sectional survey	A total of 1615(58.7%) of women in Nigeria and 979 (32.1%) in Kenya obtained short acting contraceptive methods including oral contraceptive pills, emergency contraceptives, injectables and condoms from pharmacies or drug shops.	Level D
Patrick O. eta al. 2011	Nigeria	To evaluate community pharmacists' involvement in the treatment of malaria.	Malaria	Descriptive	Cross sectional survey	The result reported little involvement of community pharmacists on preventive services such as malaria awareness, promoting the use of insecticide treated nets, and use of prophylactic drugs against malaria, with a score of 15.27 ±5.63 which is above the critical point of 15. They have made	Level D

						notable impact on curative services including ways of detecting early signs and symptoms, and rational use of anti-malarial, with a score of 20.61±3.33 which is above the critical point of 18.	
Nelissen H. et al. 2018	Nigeria	To evaluate the feasibility of Pharmacists provided blood pressure measurements and counselling.	Hypertension	Interventional	Before/after study with no control group	Pharmacists provided blood pressure measurements and medication- and lifestyle counselling employing mHealth.<br><br>High retention in care with 236 (71%) patients returned to the pharmacy after enrolment, with 3.3 months (IQR: 2.2–5.4) median duration of activity in the mHealth-application; blood pressure change (the average change in SBP was -9.9 mmHg (SD: 18.0) and DBP -5.9 mmHg (SD: 11.4) between baseline and end line; Achieving target blood pressure increased from 24% at baseline to 56% at end line ($p < 0.001$); patients' were satisfied because of accessibility, attention, adherence and information provision.	Level C
Amorha K. et al. 2020	Nigeria	To assess the impact of pharmacist-led interventions	Asthma	Interventional	Single-blind, randomised clinical trial	Pharmacists provided patient education on asthma prevalence,	Level B

		on patients' asthma control and adherence.				asthma triggers, types of inhalers, asthma control, handling of asthma attacks, asthma reviews. A significantly better asthma control compared to usual care at 3 months (P = 0.004) and 6 months (P = 0.003) was reported. Similarly, significantly better adherence compared to usual care at 3 months (P = 0.001) and 6 months (P<0.001) was reported	
Amadi C. et al. 2018	Nigeria	To assess the knowledge of cardiovascular disease (CVD) risk factors and practice of primary prevention of CVD by community pharmacists	Hypertension, Diabetes, Obesity and weight management, Dyslipidaemia	Descriptive	Cross sectional survey	Community pharmacists were reported to have involvement in routine measurement of blood pressure, blood glucose, BMI/waist circumference, advising hypertensives or diabetics to check their cholesterol level, advising hypertensives or diabetics on lifestyle management.	Level D
Adje D. et al. 2019	Nigeria	To assess outcome of cardiovascular risk assessment among rural community dwellers.	Hypertension, Dyslipidemia, Diabetes, Asthma, Obesity and weight management, Physical activity, Smoking, Alcohol use	Descriptive	Cross sectional survey	Out of rural dwellers presented to community pharmacies smoking identified in 18 (3.1%), alcohol intake in 228, (45.1%), physically inactive in 174(34.5%), high systolic and diastolic blood pressure in 232(45.9%) and 335(66.4%), abnormal blood glucose in 19	Level D

						(3.8%), abnormal cholesterol in 140(29.7%), high BMI in 247(48.9), high waist circumference in 191(37.8), and asthma in 1 (0.2).	
Abubakar U. eta al. 2019	Nigeria	To evaluate the impact of antibiotic stewardship interventions on compliance with surgical antibiotic prophylaxis practice in obstetrics and gynaecology surgeries.	Antimicrobial resistance	Interventional	Before/after study with no control group	Pharmacists were delivered antibiotic stewardship interventions in the form of developing and disseminating protocol, educational meeting, auditing and provision of feedbacks. Compliance with timing was increased from 14.2% to 43.3% (P < 0.001). Compliance with duration was also improved from 0% to 21.8% (P < 0.001). Prescription of third generation cephalosporin was reduced from 29.2% to 20.6% (P = 0.032). The rate of redundant antibiotic prescription was reduced by 19.1% (P < 0.001).	Level C
Adje D. et al. 2020	Nigeria	To assess the impact of pharmacists-initiated health education intervention on anaemia in pregnancy	Anemia	Interventional	Before/after study with control group	Pharmacists offered health education in four major areas for the intervention group. These include adherence to routine medications and scheduled antenatal appointments, healthy diet, personal and environmental hygiene, and the importance of	Level C

						using Insecticide-treated nets. Anemia prevalence was decreased among the intervention group from 44(31.4%) to 21(15.2%) compared with 55(39.3%) to 44(31.4%) in the control who received usual care ($p < 0.05$).	
Bello S. et al. 2013	Nigeria	To assess the impact of rural community pharmacist interventions on self-medications and disease prevalence.	Hypertension, Dyspepsia, Osteoarthritis, Anemia, Insomnia	Descriptive	Cross sectional survey	The result reported that community pharmacists provided adequate drug information or counselling on appropriate health management strategies of hypertension, osteoarthritis, dyspepsia, and anemia. Additionally, they have measured clients' body weight, height, body temperature and blood pressure. The intervention offered had reduced blood pressure from 161/104 to 129/86 ($P<0.05$), dyspepsia cases from 220 to 53 ($P<0.05$), and insomnia cases from 42 patients to 5 ($P<0.05$).	Level D
Amadi C. et al. 2020	Nigeria	To study the effectiveness of opportunistic screening for cardiovascular risk factors in community	Obesity, Diabetes, Hypertension, Dyslipidemia, Smoking, Alcohol use	Descriptive	Cross sectional survey	Out of 889 subjects presented to community pharmacies for screening and referral, smoking identified in 4.3%, alcohol intake in 26.7%, overweight in 59.7%, obesity	Level D

		pharmacies on apparently healthy individuals.				(BMI ≥ 30 kg/m2) in 71.5%, hypertension in 55.1%, diabetes in 3%, dyslipidemia in 45.3%, and 64.1% of the subjects were diagnosed with cardiovascular risk factors for the first time.	
Brink A. et al. 2016	South Africa	To assess the reduction of overall antibiotic consumption across a diverse group of 47 urban and rural hospitals in South Africa through the implementation of an antimicrobial stewardship strategy that uses existing resources.	Antimicrobial resistance	Interventional	Before/after study with control group	Pharmacists audited antimicrobial therapy against five measures. These include inappropriate duplicate antimicrobial coverage, no cultures were obtained before antimicrobial therapy, duration of antimicrobial therapy for greater than 7 or 14 days, patients on four or more antimicrobial agents. This intervention reduced mean antibiotic defined daily doses per 100 patient days from 101.38 (95% CI 93·05–109·72) in the pre-implementation phase to 83.04 (74·87–91·22) in the post-implementation phase (p<0·0001).	Level C
Rampamba E. et al. 2019	South Africa	To evaluate the impact of a pharmacist-led patient counselling and education model to empower hypertensive patients on	Hypertension	Interventional	Quasi-experimental design	Pharmacists delivered a hypertension information diary for daily use, with 15–30 minutes of patient counselling and education about hypertension and its management, and the correct use	Level C

		chronic medication.				of the diary. A 34.7% improvement in patients' understanding of what normal blood pressure (BP) is in the intervention group compared to the control group ($P < 0.001$), a 9.1% improvement of knowledge in the intervention group about the importance of both systolic BP and diastolic BP in controlling hypertension, post intervention, 40.0% of patients in the intervention group versus 17.9% in the control group had adequate knowledge (≥75% correct answers) about hypertension and its management, and majority (>90%) satisfied with the pharmacist intervention.	
Messina A. et al. 2015	South Africa	To evaluate the change in compliance with administering antimicrobials within an hour of prescription after implementation of a national antibiotic stewardship pharmacist-driven hang-time process	Antimicrobial resistance	Interventional	Before/after study with no control group	Pharmacists reviewed antimicrobial therapy for basic interventions such as duration of therapy greater than 7 or 14 days, inappropriate duplicate antimicrobial coverage and if cultures were obtained prior to antimicrobial therapy, and hang time compliance. Over the 60-weeks period, 21,069 patients received	Level C

		improvement protocol.				antibiotics within an hour following prescription and were assessed as hang-time compliant. Pharmacist-driven hang-time process improvement protocol was 41.2% pre-intervention week 1 (164/398) to 78.4% post-intervention week 60 (480/612; P<0.0001).	
Brink A. et al. 2016	South Africa	To implement an improvement model for perioperative antibiotic prophylaxis and achieve a reduction in surgical site infections (SSI).	Antimicrobial resistance	Interventional	Before/after study with no control group	Pharmacists were prospectively audited and provided feedback on antimicrobial prophylaxis. In total 24 206 surgical cases were reviewed. There was a significant improvement in compliance with all process measures from 66.8% (95% CI 64.8–68.7) to 83.3% (95% CI 80.8–85.8), representing a 24.7% increase (P < 0.0001). The SSI rate decreased by 19.7% from a mean group rate of 2.46 (95% CI 2.18–2.73) pre-intervention to 1.97 postintervention (95% CI 1.79–2.15) (P = 0.0029).	Level C
Mohamed S. et al. 2013	Sudan	To evaluate attitude of community pharmacists towards health education, promotion and screening, assess the	Smoking, Nutrition, Diabetes, Hypertension, Physical activity, Dyslipidemia, Obesity and weight management Oral health,	Descriptive	Cross sectional survey	Community pharmacists reported to have involved in smoking cessation activities, counselling on exercise, healthy diet, normal blood cholesterol maintenance,	Level D

		extent of their actual involvement in such activities and identify barriers for their provision.	Family planning, STIs, Unused medicine management			obesity and weight reduction, oral health, contraception methods, HIV/AIDS awareness and prevention, unused or waste medicines, and measuring of blood pressure and glucose.	
Mohamed S. et al. 2014	Sudan	To describe the current and potential roles of Sudanese community pharmacists in responding to symptoms, chronic diseases management and identify perceived barriers.	Pneumonia, Diarrhoea	Descriptive	Cross sectional survey	Community pharmacists asked the patient about his/her symptoms, provided medicines without prescription for minor diseases such as cough/pneumonia, diarrhoea, and referred to the physicians for further check-up.	Level D
Michael D. et al. 2016	Tanzania	To determine whether private drug retail outlets would be a feasible and acceptable venue to screen individuals for hypertension and subsequently direct them to proper care and treatment.	Hypertension	Descriptive	Cross sectional survey	Out of 971 customers presented to private drug retail outlets for screening and referral, 109 (11.2%) had high blood pressure and were referred for ongoing assessment, 85/109 (78.0%) were newly diagnosed, customers reported that the service was acceptable.	Level D
Maloney K. et al. 2017	Tanzania	To investigate whether the introduction of RDTs into Accredited Drug Dispensing Outlets (ADDOs) under	Malaria	Interventional	Before/after study with control group	Dispensers provided a parasite-based diagnosis using RDT test, and malaria treatment. Diagnostic uptake increased from 19% to 74% in the intervention districts and from	Level C

		realistic market conditions would improve case management practices.				3 to 18% in the control district (p < 0.01 for both groups). Among RDT positives, 90% (95% CI 81.9–94.9%) provided an anti-malarial agent, and of which 75% (95% CI 65.5–83.5%) received an artemisinin combination therapy.	
Kahabuka C. et al. 2013	Tanzania	To assess care-seeking and management of suspected malaria, pneumonia and diarrhoea at various health care facilities	Malaria, Pneumonia Diarrhoea	Descriptive	Cross sectional survey	A total of 344 (23.2%) fever, 157 (20.8%) diarrhoea, and 126 (26.3) acute respiratory infection (pneumonia) cases were treated by artemisinin combination therapy, quinine, fansider, chloroquine or amodiaquine, oral rehydration salts or home rehydration solution and antibiotics in private pharmacies. No data for pneumonia.	Level D
Rutta E. et al. 2014	Tanzania	To assess the level of knowledge about tuberculosis (TB), practices related to identification of patients with suspected TB, the availability of educational materials, training, and treatment.	Tuberculosis	Descriptive	Cross sectional survey	Out of 295 dispensers, 280 (95%) identified persistent cough as a symptom of TB, 8% of outlets stocked first-line anti-tuberculosis medicines. The majority of dispensers reported seeing clients with TB-like symptoms, and of these 95% reported frequently referring clients to nearby health facilities.	Level D

Kitutu F. et al. 2017	Uganda	To determine the effect of integrated community case management intervention (iCCM) derived from the WHO/UNICEF recommended iCCM on appropriate testing and treatment for uncomplicated malaria, pneumonia symptoms and non-bloody diarrhoea among under 5-year children	Malaria, Pneumonia, Diarrhoea	Interventional	Quasi-experimental design	The reported intervention improved uptake of diagnostic testing such as use of thermometer, malaria RDTs and use of respiratory timers by 41.2% (95% CI 19.4, 63.0), 52.6% (95% CI 27.3, 77.9) and 60.1% (95% CI 47.6, 72.6) respectively. Similarly, the intervention increased the appropriate treatment of uncomplicated malaria, pneumonia symptoms and non-bloody diarrhoea by 80.2% (95% CI 53.2–107.2), 65.5% (95% CI 51.6–79.4) and 31.4% (95% CI 1.6–61.2), respectively.	Level C
Akol A A. et al. 2014	Uganda	To assess the contribution of drug shops to family planning service provision.	Family planning	Descriptive	Cross sectional survey	Drug shop operators (DSO) counselled and administered family planning methods such as depot medroxyprogesterone acetate (DMPA) injections, implants, condoms and oral contraceptive pills for 585 clients. Majority of clients 94% continue to go to DSO for family planning services, 99% satisfied with the service, 100% treated with respect and 93% trusted the DSO to protect the privacy.	Level D

Awor P. et al. 2014	Uganda	To assess the feasibility and effect on access and appropriateness of treatment when we introduce diagnostics (rapid diagnostic tests (RDTs) and respiratory timers) and promote pre-packaged paediatric-dosage drugs for acute febrile illnesses (malaria and pneumonia) and diarrhoea.	Malaria, Pneumonia Diarrhoea	Interventional	Quasi-experimental design	In drug shops malaria was diagnosed using rapid diagnostic tests (RDTs) and artemisinin combination therapy dispensed in 427 (87.7%) and 343(70.4%) respectively; respiratory timer used and amoxicillin dispensed in 40/73 (54.8%) and 36/73 (49.3%) respectively; children with diarrhoea were 176(35.4%) and ORS and zinc dispensed in 136 (77.3%).	Level C

1. International Pharmaceutical Federation (FIP). FIP Statement of Policy the role of the pharmacist in the prevention and treatment of chronic disease, approved by FIP Council, Brazil 2006 [Available from: https://www.fip.org/file/4338.

2. World Bank. World Bank Global Index LMIC List 2020 [Available from: https://data.worldbank.org/income-level/low-and-middle-income.

3. World Health Organization (WHO). Noncommunicable diseases. 2021 [Available from: https://www.who.int/news-room/fact-sheets/detail/noncommunicable-diseases.

4. Bello SI, Bello IK. Impacts of community pharmacists on self-medication management among rural dwellers, Kwara State Central, Nigeria. 2013.

5. Amadi C, Lawal F, Ajiboye W, Agbim R, Mbakwem A, Ajuluchukwu J, et al. Opportunistic screening of cardiovascular disease risk factors in community pharmacies in Nigeria: a cross-sectional study. 2020;42(6):1469-79.

6. Nelissen HE, Cremers AL, Okwor TJ, Kool S, van Leth F, Brewster L, et al. Pharmacy-based hypertension care employing mHealth in Lagos, Nigeria–a mixed methods feasibility study. 2018;18(1):1-14.

7. Adje DU, Williams FE, Oparah ACJAhs. Outcome of cardiovascular risk assessment among rural community dwellers in ezionum, Nigeria-implications for public health. 2019;19(2):2112-20.

8. Rampamba EM, Meyer JC, Helberg EA, Godman BJ. Empowering hypertensive patients in South Africa to improve their disease management: a pharmacist-led intervention. Journal of research in pharmacy practice. 2019;8(4):208.
9. Amadi CE, Lawal FO, Mbakwem AC, Ajuluchukwu JN, Oke DA. Knowledge of cardiovascular disease risk factors and practice of primary prevention of cardiovascular disease by Community Pharmacists in Nigeria: a cross-sectional study. J International journal of clinical pharmacy. 2018;40(6):1587-95.
10. Michael D, Kezakubi D, Juma A, Todd J, Reyburn H, Renju JJIh. The feasibility and acceptability of screening for hypertension in private drug retail outlets: a pilot study in Mwanza region, Tanzania. 2016;8(5):360-6.
11. Marfo AF, Owusu-Daaku FTJIJoPP. Evaluation of a pharmacist-led hypertension preventative and detection service in the Ghanaian community pharmacy: an exploratory study. 2016;24(5):341-8.
12. Mohamed SS-E, Mahmoud AA, Ali AAJIjocp. Involvement of Sudanese community pharmacists in public health activities. 2013;35(3):393-400.
13. Erku DA, Mersha AGJPo. Involvement of community pharmacists in public health priorities: A multi-center descriptive survey in Ethiopia. 2017;12(7):e0180943.
14. Tesfaye ZT, Yismaw MBJPo. Community's extent of use and approval of extended pharmacy services in community pharmacies in Southwest Ethiopia. 2020;15(4):e0230863.
15. Belachew SA, Muluneh NY, Erku DA, Netere AKJPo. A cross sectional study on beliefs and roles of community pharmacy professionals in preventing and managing metabolic syndrome in an Ethiopian setting. 2020;15(12):e0244211.
16. Teka NT, Baye AMJBrn. Counseling practice of community pharmacists for diabetes mellitus patients in Addis Ababa, Ethiopia. 2018;11(1):1-4.
17. Adje D, Umar M, Gadanya M, Rufai R. IMPACT OF PHARMACISTS'HEALTH EDUCATIONAL INTERVENTION ON ANEMIA IN PREGNANCY IN RURAL SETTINGS IN KANO, NIGERIA. 2020.
18. Amorha KC, Okonta MJ, Ukwe CVJIJoCP. Impact of pharmacist-led educational interventions on asthma control and adherence: single-blind, randomised clinical trial. 2020:1-9.
19. World Health Organization (WHO). Communicable Diseases. [Available from: https://www.afro.who.int/health-topics/communicable-diseases#:~:text=Communicable%2C%20or%20infectious%20diseases%2C%20are%20caused%20by%20microorganisms,are%20caused%20by%20ingesting%20contaminated%20food%20or%20water.
20. Patrick OK, Joshua AK, Oluwafunmi A. Evaluation of community pharmacists' involvement in the treatment of malaria in Nigeria. International Journal of Pharmacy and Pharmaceutical Sciences. 2011;3:107-12.
21. Maloney K, Ward A, Krenz B, Petty N, Bryson L, Dolkart C, et al. Expanding access to parasite-based malaria diagnosis through retail drug shops in Tanzania: evidence from a randomized trial and implications for treatment. 2017;16(1):1-10.
22. Kitutu FE, Kalyango JN, Mayora C, Selling KE, Peterson S, Wamani HJMj. Integrated community case management by drug sellers influences appropriate treatment of paediatric febrile illness in South Western Uganda: a quasi-experimental study. 2017;16(1):1-18.

23. Kahabuka C, Kvåle G, Hinderaker SGJPO. Care-seeking and management of common childhood illnesses in Tanzania–results from the 2010 Demographic and Health Survey. 2013;8(3):e58789.
24. Awor P, Wamani H, Tylleskar T, Jagoe G, Peterson SJPO. Increased access to care and appropriateness of treatment at private sector drug shops with integrated management of malaria, pneumonia and diarrhoea: a quasi-experimental study in Uganda. 2014;9(12):e115440.
25. Poyer S, Musuva A, Njoki N, Okara R, Cutherell A, Sievers D, et al. Fever case management at private health facilities and private pharmacies on the Kenyan coast: analysis of data from two rounds of client exit interviews and mystery client visits. 2018;17(1):1-17.
26. Ansah EK, Narh-Bana S, Affran-Bonful H, Bart-Plange C, Cundill B, Gyapong M, et al. The impact of providing rapid diagnostic malaria tests on fever management in the private retail sector in Ghana: a cluster randomized trial. 2015;350.
27. Kwarteng A, Malm KL, Febir LG, Tawiah T, Adjei G, Nyame S, et al. The Accuracy and Perception of Test-Based Management of Malaria at Private Licensed Chemical Shops in the Middle Belt of Ghana. 2019;100(2):264.
28. Mohamed SS, Mahmoud AA, Ali AAJIjocp. The role of Sudanese community pharmacists in patients' self-care. 2014;36(2):412-9.
29. Ayele AA, Mekuria AB, Tegegn HG, Gebresillassie BM, Mekonnen AB, Erku DAJPo. Management of minor ailments in a community pharmacy setting: findings from simulated visits and qualitative study in Gondar town, Ethiopia. 2018;13(1):e0190583.
30. Mengistu G, Gietnet K, Amare F, Sisay M, Hagos B, Misganaw DJCMIP. Self-reported and actual involvement of community pharmacy professionals in the management of childhood diarrhea: a cross-sectional and simulated patient study at two towns of Eastern Ethiopia. 2019;13:1179556519855380.
31. Rutta E, Tarimo A, Delmotte E, James I, Mwakisu S, Kasembe D, et al. Understanding private retail drug outlet dispenser knowledge and practices in tuberculosis care in Tanzania. 2014;18(9):1108-13.
32. World Health Organization (WHO). Sexual and Reproductive Health Fact Sheet. 2017 [Available from: https://www.afro.who.int/publications/sexual-and-reproductive-health-fact-sheet.
33. Gonsalves L, Wyss K, Gichangi P, Hilber AMJIjoph. Pharmacists as youth-friendly service providers: documenting condom and emergency contraception dispensing in Kenya. 2020;65:487-96.
34. Akol A, Chin-Quee D, Wamala-Mucheri P, Namwebya JH, Mercer SJ, Stanback JJGHS, et al. Getting closer to people: family planning provision by drug shops in Uganda. 2014;2(4):472-81.
35. Corroon M, Kebede E, Spektor G, Speizer IJGHS, Practice. Key role of drug shops and pharmacies for family planning in urban Nigeria and Kenya. 2016;4(4):594-609.
36. Lebetkin E, Orr T, Dzasi K, Keyes E, Shelus V, Mensah S, et al. Injectable contraceptive sales at licensed chemical seller shops in Ghana: access and reported use in rural and periurban communities. 2014;40(1):21-7.

37. Liambila W, Obare F, Keesbury JJPe, counseling. Can private pharmacy providers offer comprehensive reproductive health services to users of emergency contraceptives? Evidence from Nairobi, Kenya. 2010;81(3):368-73.
38. Gonsalves L, Wyss K, Gichangi P, Say L, Martin Hilber AJPo. Regulating pharmacists as contraception providers: a qualitative study from coastal Kenya on injectable contraception provision to youth. 2019;14(12):e0226133.
39. Mugo PM, Duncan S, Mwaniki SW, Thiong'o AN, Gichuru E, Okuku HS, et al. Cross-sectional survey of treatment practices for urethritis at pharmacies, private clinics and government health facilities in coastal Kenya: many missed opportunities for HIV prevention. 2013;89(7):583-9.
40. Mugo PM, Prins HA, Wahome EW, Mwashigadi GM, Thiong'o AN, Gichuru E, et al. Engaging young adult clients of community pharmacies for HIV screening in Coastal Kenya: a cross-sectional study. 2015;91(4):257-9.
41. World Health Organization (WHO). Antimicrobial resistance 2020 [Available from: https://www.who.int/news-room/fact-sheets/detail/antimicrobial-resistance.
42. Brink AJ, Messina AP, Feldman C, Richards GA, Becker PJ, Goff DA, et al. Antimicrobial stewardship across 47 South African hospitals: an implementation study. 2016;16(9):1017-25.
43. Messina AP, van den Bergh D, Goff DAJID, Therapy. Antimicrobial stewardship with pharmacist intervention improves timeliness of antimicrobials across thirty-three hospitals in South Africa. 2015;4(1):5-14.
44. Brink AJ, Messina AP, Feldman C, Richards GA, van den Bergh D, Chemotherapy NASSAJJoA. From guidelines to practice: a pharmacist-driven prospective audit and feedback improvement model for peri-operative antibiotic prophylaxis in 34 South African hospitals. 2017;72(4):1227-34.
45. Abubakar U, Syed Sulaiman SA, Adesiyun AGJPo. Impact of pharmacist-led antibiotic stewardship interventions on compliance with surgical antibiotic prophylaxis in obstetric and gynecologic surgeries in Nigeria. 2019;14(3):e0213395.
